# Brightness-gated two-color coincidence detection unravels two distinct mechanisms in bacterial protein translation initiation

**DOI:** 10.1038/s42003-019-0709-7

**Published:** 2019-12-06

**Authors:** Henning Höfig, Olessya Yukhnovets, Cristina Remes, Noemie Kempf, Alexandros Katranidis, Daryan Kempe, Jörg Fitter

**Affiliations:** 1 I. Physikalisches Institut (IA), RWTH Aachen University, Aachen, Germany; 2 Institute of Complex Systems ICS-5, Forschungszentrum Jülich, Jülich, Germany; 3 Present Address: Max Planck Institute for the Biology of Ageing, Cologne, Germany; 4 Present Address: Laboratoire de Biologie Moléculaire Eucaryote LBME—Center for Integrative Biology CBI, University of Toulouse, Toulouse, France; 5 Present Address: EMBL Australia, Single Molecule Science Node, School of Medical Sciences, University of New South Wales, Sydney, NSW Australia

**Keywords:** Biochemical assays, Single-molecule biophysics

## Abstract

Life on the molecular scale is based on a complex interplay of biomolecules under which the ability of binding is crucial. Fluorescence based two-color coincidence detection (TCCD) is commonly used to characterize molecular binding, but suffers from an underestimation of coincident events. Here, we introduce a brightness-gated TCCD which overcomes this limitation and benchmark our approach with two custom-made calibration samples. Applied to a cell-free protein synthesis assay, brightness-gated TCCD unraveled a previously disregarded mode of translation initiation in bacteria.

## Abstract (web summary)

Henning Höfig et al. present a brightness-gated two-color coincidence detection method that overcomes the limitations of confocal detection. The method adds a selection criterion for the brightness of single fluorescence bursts, substantially improving the counting accuracy for single fluorophores.

## Introduction

Numerous cellular processes, including biochemical signaling or the assembly of higher order complexes such as ribosomes, are regulated through the binding affinities of several interaction partners^1–3^. Traditionally, these are determined by biochemical/biophysical techniques like electrophoresis^4^, surface plasmon resonance^5^, or isothermal titration calorimetry^6^. In recent years, due to their high specificity, sensitivity, as well as applicability, fluorescence based methods have become increasingly important^7,8^.

By attaching fluorophores of different colors to each of the binding partners, simultaneous dual-color fluorescence detection techniques can be employed to characterize binding interactions. For surface-tethered molecules, co-localization of both colors using wide-field or total internal reflection fluorescence microscopy is a common approach, not only to validate intermolecular binding, but also to determine the stoichiometry of subunits in larger molecular complexes^9–11^. Although the cover-slip surfaces are passivated to prevent unspecific binding, interactions between them and the anchor sequence or the molecule itself cannot be ruled out completely and might affect binding characteristics. Bimolecular binding of freely diffusing fluorescent molecules is frequently studied by Förster resonance energy transfer (FRET)^12,13^ or fluorescence cross-correlation spectroscopy (FCCS)^14^. While FCCS allows to determine concentrations of unbound and bound species in a solution, the accuracy of the obtained results often depends on multiple calibration steps and the pre-selection of a fitting model^15^.

Two-color coincidence detection (TCCD)^16–18^ is probably the most straightforward approach to quantify the fraction of bound and unbound molecules in an ensemble of diffusing molecules. TCCD utilizes the dual-color confocal detection of single molecules and a subsequent coincidence analysis of both fluorescence signals that reveals whether the interaction partners are associated or not. TCCD is conceptually related to single-molecule FRET (smFRET) measurements that use alternating laser excitation (ALEX)^19^ or pulsed interleaved excitation (PIE)^20^ to facilitate the calculation of stoichiometry ratios. TCCD does not require pulsed excitation, but it can still be applied to smFRET data to determine the amount of single and dual-labeled molecules. However, a mismatch between the two confocal volumes, temporal fluctuations of the fluorescence emission, and distinct differences between the emission rates of both fluorophores often result in a pronounced underestimation of coincidence in TCCD analyses. The utilization of cylindrical illumination in combination with microfluidics offers an increased overlap of the detection volumes but has a reduced single fluorophore sensitivity^21^. Microfluidic devices can also funnel the particle transits through the center of confocal detection volume and allow furthermore for a larger flow rate which increases the rate of data aquisition^22^. However, sample aggregation and surface interactions within the microfluidic channels can limit the application.

Here, we present a Brightness-gated two-color coincidence detection (BTCCD) that overcomes above mentioned limitations in standard confocal detection. We first demonstrate the performance of BTCCD by means of two custom-designed DNA-based calibration samples and define optimal brightness gates for obtaining correct results. Finally, we apply BTCCD to monitor the subunit dissociation of 70S ribosomes for translation initiation in a cell free transcription/translation assay and find evidence suggesting a previously undetermined second mechanism in bacterial protein synthesis initiation. According to our current understanding, protein synthesis initiation of canonical mRNA requires dissociated 30S and 50S ribosomal subunits before translation and nascent chain elongation can start^23^. In particular, the initiation requires a pool of free 30S and 50S ribosomal subunits, a mRNA with a Shine-Dalgarno (SD) sequence (canonical mRNA) and three initiation factors (IFs). This mechanism postulates that the first initiation step is the binding of mRNA SD-sequence to the 30S subunit together with some initiation factors. The obtained 30S-initiation complex has a high affinity to 50S subunits and both together form a 70S complex, which then enters the elongation phase of the translation process. Our use of BTCCD suggests the possibility that initiation of protein synthesis can also take place directly by 70S complexes, i.e. 30S and 50S subunits remain associated.

## Results

### Brightness-gated two-color coincidence detection

**Fig. 1:**
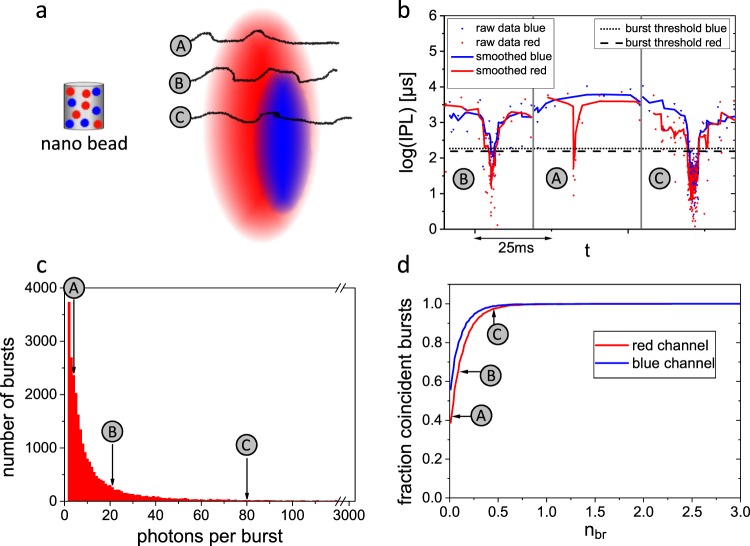
Concept of brightness-gated two-color coincidence detection is based on excluding non-central molecule trajectories. Data shown for 100% dual-labeled nano-bead reference.**a** Mismatching confocal volumes represented by red and blue ellipsoids. Three classes of trajectories of diffusing molecules are depicted as black lines: borderline (A), peripheral (B), and central (C) with respect to centroid of red ellipsoid. Note, that the nano-bead is not on scale. **b** Segments of inter-photon lag (IPL) time trace. Dips of the IPLs below the burst thresholds reflect single-molecule transits that correspond to trajectories (A), (B), and (C) illustrated in **a**. **c** Histogram of number of photons per burst for red channel (Atto 647N). Borderline trajectories (A) with a low number of photons per burst are much more abundant than central trajectories (C) with a high number of photons per bursts. **d** Fraction of coincident bursts as a function of normalized brightness threshold *n*_br_. Bursts with a low brightness threshold include all trajectories (A), central molecule trajectories (C) reach the expected value of 100% coincidence. Burst and analysis statistics are given in Supplementary Note 1.

The coincidence analysis is based on single-molecule data obtained by dual-color confocal detection of diffusing fluorescent molecules. The BTCCD approach utilizes two different thresholds for the fluorescence signal of each color: the burst threshold is used to discriminate a single-molecule event against the background^24^ and the brightness threshold selects bursts that exceed a certain brightness. For each color, the coincidence of bright bursts to all bursts of the other color is determined (see Supplementary Fig. 1 for flow chart). Due to the shorter wavelength, the blue excitation light produces a smaller diffraction limited detection volume as compared to the red excitation light. In addition, lens aberrations in the high NA objective typically cause a small spatial shift between the detection volumes of both colors. Figure 1a illustrates the resulting incomplete overlap of the confocal volumes. Molecule trajectories that just slightly touch one of the confocal volumes (trajectory A and B in Fig. 1a) do not result in bright fluorescence bursts and are not considered for analysis. In contrast, a molecule that takes a central trajectory through the volume of one color (trajectory C in Fig. 1a) will generate a burst that is very bright in this color and will most likely also penetrate the volume of the other color. Additionally, bright bursts correspond to large dwell times in the volumes, which make it likely that transitions from a potential dark state (e.g. triplet state) back to the bright state occur during the observation time.

### Nano-bead calibration

First, we investigated the effect of a confocal volume mismatch on the obtained coincidence. To resemble the spatial profile of a fluorophore pair attached to a (bio)molecule (complex), we used a custom-designed nano-bead DNA-origami structure carrying a distribution of 5–10 dye copies of each fluorophore (Alexa 488 and Atto 647N) at a distance of approximately 10 nm. By labeling each molecule with multiple dye copies we ensure 100% dual-labeling of all molecules and exclude a full darkening because the dyes blink independently.

Due to the stochastic character of the diffusion process (random walk) many different trajectories through the confocal detection volumes occur (Fig. 1a). When a molecule transits through the volumes it will emit a burst of fluorescence photons. Each burst appears as a dip in the inter-photon lag (IPL) and is detected by means of a burst threshold (dotted and dashed line in Fig. 1b for blue and red channel, respectively). The distribution of the number of photons per burst for the red channel is depicted in Fig. 1c. Central trajectories (trajectory C) that correspond to a high number of photons per burst are rarely observed. In contrast, borderline and peripheral trajectories (trajectory A and B, respectively) that correspond to bursts with a small to medium number of photons are rather frequent. The same observations can be made for the blue channel (see Supplementary Fig. 2a). If a borderline trajectory is followed (trajectory A), the IPL time trace of the red channel drops below the burst threshold, whereas the IPL time trace of the blue channel does not (see solid lines in Fig. 1b). As a consequence, no coincidence is detected although the molecule is in fact dual labeled. In the case of a peripheral trajectory (trajectory B), it is possible but not certain that the molecule is detected in the blue channel to confirm the coincidence. Only a central trajectory through both detection volumes leads to a pronounced burst in both detection channels, meaning that only bursts whose number of photons exceeds a certain brightness threshold should be considered for the coincidence analysis.

Figure 1d shows how the fraction of coincident bursts evolves when the brightness threshold *n*_br_ (normalized to the mean number of photons per burst) is increased. The curves for both colors are depicted, as the brightness threshold can either be applied to the red channel and the coincidence to all burst in the blue channel is evaluated or vice versa. Both curves show a similar trend, i.e. a steep increase of coincidence for low brightness thresholds followed by a plateau for larger brightness thresholds. The coincidence fraction of the red channel starts at a value of ~40%, which means that only 40% of the trajectories within the red volume do additionally penetrate the blue volume. This is a direct consequence of the larger red confocal volume. Accordingly, the blue coincidence fraction starts at a higher value of ~55%. The fact that the initial blue coincidence fraction deviates from 100% confirms a relative shift between both volumes (see Supplementary Fig. 3). The same reasoning explains why the blue coincidence saturates at lower brightness thresholds than the red coincidence. However, only for brightness thresholds exceeding a value of 0.75 both coincidence fractions reach the expected value of 100% which underlines the need to brightness-gate TCCD.

### Single dye pair calibration

**Fig. 2:**
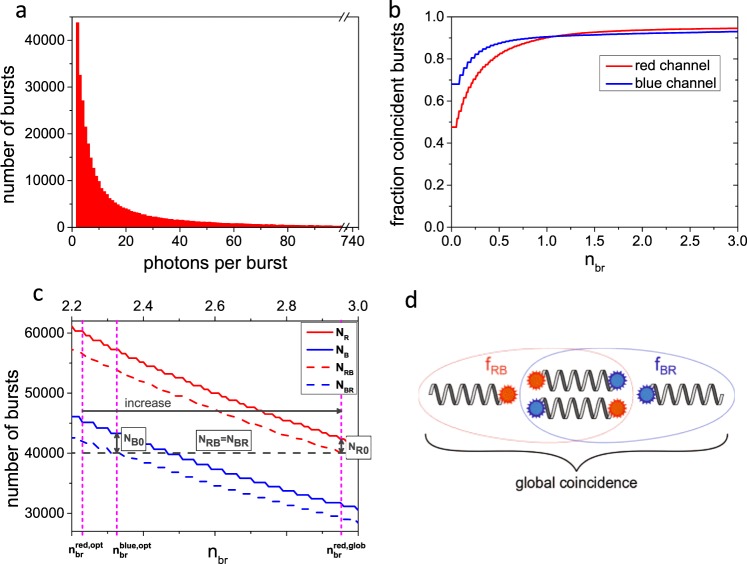
Benchmark of brightness-gated two-color coincidence detection with single dye pair dsDNA reference. **a** Histogram of number of photons per burst for red channel (Atto 647N). **b** Fractions of coincident bursts as a function of normalized brightness threshold *n*_br_. Obtained saturated coincidence fractions are 0.94 and 0.92 for red and blue channel, respectively. Burst and analysis statistics are given in Supplementary Notes 1 and 2. **c** Concept of global coincidence analysis. Number of selected red and blue bursts (solid lines) and those which show coincidence with the complementary color (dashed lines) are shown as a function of brightness threshold *n*_br_. The optimal brightness threshold *n*_br,opt_ is depicted for each color separately (see Supplementary Note 5). The brightness threshold of the color with the higher number of coincident bursts is increased (here red) until the number of coincident bursts are equal, i.e. *N*_RB_ = *N*_BR_ as indicated by the gray dashed line. The number of red-only and blue-only bursts *N*_*R0*_ and *N*_*B0*_, respectively, are indicated by arrows. **d** Differentiation between color-specific coincidence fractions and global coincidence.

Next, we investigated the impact of single dye fluorescence emission on the coincidence because most experimental assays rely on single dye labeling. For this purpose, we custom-designed a double stranded (ds) DNA molecule which was aimed to be completely labeled with a pair of Alexa 488 and Atto 647N. Incomplete dual-labeling due to potential dsDNA dissociation was minimized by attaching both dyes to the same strand (see Methods). The obtained results are summarized in Fig. 2.

The distribution of photons per burst is similar to that of the nano-bead sample (cf. Figure 2a and Fig. 1c, match obtained intentionally by adjusting laser power). However, the coincidence fractions as a function of the brightness threshold (cf. Figs. 1d and 2b) exhibit differences in the y-intercept (without brightness threshold, i.e. *n*_br_ = 0), which we attribute to the smoothing of the IPL time trace and different burst thresholds. Nevertheless, a similar steep increase for brightness thresholds lower than 0.8 is observed which turns into a shallow plateau for larger brightness thresholds. The determined coincidence ratios are 94 ± 1% and 92 ± 1% for the red and the blue channel, respectively, and show a slight deviation from full coincidence attributed to certain incompleteness of the labeling. In addition, we performed multi-point calibrations with mixtures of different fractions of single and double-labelled dsDNA (see Supplementary Note 3). The results demonstrate the high reliability in determining coincidence fractions over the whole range of possible values.

To evaluate the effect of long-lived (ms-s) dark states on the outcome of our BTCCD, we exchanged Atto 647N to Alexa 647, a dye known to exhibit pronounced photo-switching^25^. In this case the determined coincidence ratios are 95 ± 1% and 79 ± 3% for the red and the blue channel, respectively (see Supplementary Fig. 4). The coincidence values were robust against moderate laser power variations and showed a partial reduction at higher laser powers (see Supplementary Note 4).

### Global coincidence analysis

For a global coincidence analysis, i.e. to determine the number of only-blue (*N*_B0_), only-red (*N*_R0_) and dual-labeled (*N*_RB_,*N*_BR_) molecules in an ensemble, we use the optimal brightness thresholds of the individual channels as initial parameters (see Supplementary Note 5 for determination of optimal brightness thresholds). These optimal brightness thresholds define the number of selected bursts *N*_*B*_, *N*_*R*_ and the number of coincident bursts *N*_BR_, *N*_RB_ in the blue and red channel, respectively. As the numbers of coincident bursts at the respective optimal brightness threshold are in general not equal, we increase the brightness threshold of the channel that exhibits the higher number of coincident bursts until *N*_BR_ and *N*_RB_ are equal. This reduces the absolute number of selected bursts, but barely changes the coincidence fraction (cf. plateau in Fig. 2b). In Fig. 2c the number of bursts in the red and blue channel (solid lines) and the number of coincident bursts (dashed lines) are plotted as a function of the brightness threshold. The optimal brightness thresholds $n_{{\mathrm{br}}}^{{\mathrm{opt}}}$ are 2.34 for the blue channel and 2.23 for the red channel (Supplementary Note 5). Here, the brightness threshold of the red channel is increased until the number of coincident bursts are equal (gray dashed line), making it possible to quantify the relative abundance of all three possible species of molecules (*N*_*BR*_ = *N*_*RB*_, *N*_*B0*_, and *N*_*R0*_, see Fig. 2d and Table 1).

**Table 1:** Absolute number of bursts and corresponding statistical weights as obtained by global coincidence analysis.

*N*_RB_, *N*_BR_	*N*_R0_	*N*_B0_	*N*_tot_
40,000	2300	3300	45,600
88%	5%	7%	100%

BTCCD can also be applied to molecular complexes which are linked to a pair of fluorescent proteins (FPs) of different colors. Such structures are often employed as FRET-based biosensors^26^ and BTCCD can characterize the amount of individual chromophore maturation, a crucial parameter within these sensor constructs (see Supplementary Note 6).

### Evidence for 70S ribosome translation initiation

Finally, we applied BTCCD to study ribosomal translation initiation in bacteria. For this purpose we employed a cell-free protein synthesis (CFPS) system, which allowed us to produce a fast maturating green fluorescent protein variant (GFP Emerald) in vitro using labeled ribosomes and canonical mRNA^11^. The ribosomes were site-specifically labeled with Cy5 dye at the 50S subunit (see Methods). GFP remained bound to the ribosome after synthesis due to a short arrest peptide sequence (for details see ref. ^11^).

**Fig. 3:**
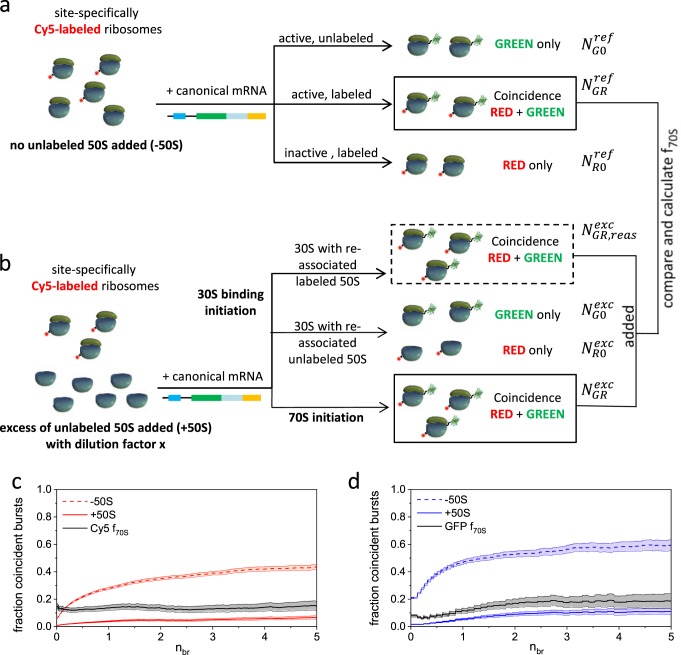
Differentiation between 30S binding and 70S initiation modes is determined in an in vitro transcription-translation fractionated system. **a** Reference reaction without excess of unlabeled 50S subunits is used to characterize the cell-free assay. Cy5 labeled ribosomes synthesize GFP which is arrested at the ribosome. Unlabeled or inactive ribosomes might be present and reduce the coincidence. **b** Same reaction as in **a** with excess of unlabeled 50S subunits. 30S binding initiation will further reduce the coincidence and, hence, comparison of both coincidence fractions will reveal the initiation mode. **c**, **d** The coincidence fractions as a function of normalized brightness threshold *n*_*br*_ are shown for assays without (−50S, dashed lines) and with 20-fold excess of unlabeled 50S subunits (+50S, solid lines). As shown for the Cy5 channel (**c**) and for the GFP channel (**d**) the corresponding coincidence ratios (*f*_70S_) level off at values ~0.14 and ~0.18, respectively (black solid lines), see sample #5 in the Supplementary Note 7 Tab. N7.1. The shaded areas give the experimental error, see Supplementary Note 1 Tab. N1.3, N1.4, N1.5 for counting statistics.

We first performed a reference synthesis to characterize the CFPS system (Fig. 3a). BTCCD can distinguish between three different scenarios taking place during synthesis: ribosomes that are active but not successfully labeled appear as green-only molecules; ribosomes that were successfully labeled but are inactive will, in contrast, appear as red-only molecules; and active ribosomes that were successfully labeled appear as red and green coincident molecules. This coincidence fraction serves as a reference because it determines the highest detectable coincidence of red labeled ribosomes that diffuse with a bound green GFP.

Performing the same synthesis with an excess of unlabeled 50S subunits (+50S) determines the fraction of ribosomes that use the 30S-binding initiation mode and the fraction that uses 70S initiation (Fig. 3b). In the case of 30S-binding initiation, dissociation of subunits occurred in order to form the 30S initiation complex. Because of the high excess of unlabeled 50S subunits, the 30S initiation complex subsequently binds predominately to an unlabeled 50S subunit. This leads to a GFP synthesized by a non-labeled 70S ribosome (green only) and likewise to a free red-labeled 50S subunit (red only). In the case of 70S initiation, the subunits did not dissociate and, hence, the reaction leads to a GFP bound to a labeled ribosome (coincidence of red and green). The comparison of the coincidence in absence and in presence of an excess of unlabeled 50S subunits reveals the probability of the respective initiation mode *p*_30S_ and *p*_70S_, respectively. A purely 30S binding initiation would theoretically lead to a total loss of coincidence and, accordingly, exclusive 70S initiation would lead to the same coincidence as the reference. Consequently, a mixture of initiation modes leads to a partly reduced coincidence. The experimentally observed coincidence fraction depends also on the excess of unlabeled 50S subunits compared to the labeled 50S subunits which are part of the functional 70S complex. Depending on the unlabeled 50S excess, there is a certain probability (*p*_reas_) for freely diffusing labeled 50S subunits (produced during the 30S initiation) to re-associate with 30S subunits (see dashed box in Fig. 3b). Exemplarily, the measured coincidence fractions as a function of the brightness threshold are shown for the red channel (i.e. Cy5 coincidence) in Fig. 3c and for the green channel (GFP coincidence) in Fig. 3d. The coincidence fractions without unlabeled 50S (−50S, dashed lines) are larger than the coincidence fractions with unlabeled 50S excess (+50S, solid lines). If the coincidence fractions of the +50S experiment are normalized to the ones of the −50S experiment, more or less constant values for *f*_70S_ are obtained (solid black lines). Typically, the data show for the obtained *f*_70S_ values a slight deviation between the GFP and the Cy5 channel. However, in total we performed seven individual reactions for which the experimental data and related results are shown in the Supplementary Note 7 (see Figs. N7.1–3 and Tab. N7.1). As discussed in the Supplementary Note 7 we can calculate the probability p_70S_ from the experimental *f*_70S_ values. A fit to all our experimental *f*_70S_ values (see Fig. N7.4) gives a value of *p*_70S_ = 0.181 ± 0.030, i.e. the protein synthesis follows with 18% the 70S initiation path and with 82% the 30S initiation. Assuming that the highest possible *p*_*reas*_ does not describe the most probable scenario, the obtained *p*_70S_ values represent a lower boundary and slightly higher values are possible (see Supplementary Note 7).

## Discussion

BTCCD differs from other TCCD methods^16,17,27,28^ through an additional selection criterion for the brightness of single fluorescence bursts, which improves the accuracy of the obtained coincidence fractions for single fluorophores down to an error level of about 5% (see Supplementary Notes 2 and 3, Fig. N2 and Fig. N3). Using custom, 100% dual-labeled nano-beads, BTCCD reaches a coincidence of 100%, whereas state-of-the-art TCCD underestimates the coincidence by 20% (see Supplementary Note 8). In the case of single dye pairs, the difference even reaches 30%, proving the superiority of BTCCD.

To reach a high level of accuracy, less photo-stable dyes with long dark states (e.g., Alexa 647, Cy5) require a specific calibration. However, if only changes between different sample conditions need to be monitored, a calibration measurement is inherently performed, as shown in the translation initiation experiments (see Fig. 3). Here, the sample without excess of 50S ribosomal subunits served as the calibration reference for the main experiment with the excess of 50S subunits.

It is also worth mentioning that nano-beads, developed as an ideal calibration sample for BTCCD, could serve as a new standard for applications where a precise knowledge of the confocal volume overlap is required. Both fluorophore clusters are placed at a well-defined distance of 10 nm which allows capturing the geometry of the confocal volumes more precisely as compared to other calibration samples, like TetraSpeck beads.

Using BTCCD, we found that the generally accepted mode of 30S binding initiation^23,29^ for bacterial protein synthesis is used by ~82% of the ribosomes. Under the conditions used in our study the remaining ~18% of ribosomes make use of a 70S initiation. Some previous studies suggested that 70S ribosomes tethered on a surface are also able to translate canonical mRNAs, indicating that dissociation and full separation of subunits and initiation with a free 30S complex is not required^30–32^. So far, cases of translation initiation which directly start with 70S complexes have only been shown for leaderless mRNAs that lack the SD sequence^33,34^. In addition, a so-called 70S scanning mode is known, in which translation of the downstream cistron in a bicistronic mRNA can be initiated by sliding of the 70S ribosome after translation of the first cistron^35^. However, here we demonstrated for the first time that also with canonical mRNA a 70S initiation takes place to a certain extent. BTCCD is a unique tool for measurement of cell free protein synthesis assays and prospective studies will intend to unravel how environmental conditions affect the distribution of different translation initiation modes.

In addition to the presented case study on the mechanisms of bacterial protein synthesis initiation, several further applications are within the scope of BTCCD. This includes for example studies on binding partners with extremely high binding affinities^36^ to determine *K*_*d*_ values, since the BTCCD approach can be applied to very low sample concentrations (below pico-molar). Another example is given by applications on molecular complexes equipped with fluorescent proteins. Here, a global BTCCD analysis can provide valuable information about the individual chromophore maturation of fluorescent proteins in FRET-based biosensors^37,38^ (see Supplementary Note 6). For absolute and quantitative readout information from genetically encoded biosensors, the level of expression and chromophore maturation of the involved FPs are extremely crucial parameters^39^.

Finally, we can summarize that the employed algorithm is robust (Supplementary Note 1), easy to implement and exhibits only minor impairments caused by photo-bleaching or by effects related to different excitation intensities (Supplementary Note 4). Thus BTCCD provides a reliable quantification tool for bound and non-bound species in the ensemble of a sample (Supplementary Note 3) with a recognizable potential for further interesting applications.

## Methods

### dsDNA reference samples

The nano-bead reference was a custom DNA-origami structure (diameter of the origami structure: 23 nm) labeled with an average number of 5–10 dye copies of each color, namely Alexa 488 and Atto 647N (purchased as GATTA-Bead RB from Gattaquant, Braunschweig, Germany). The single dye references samples were produced by hybridizing a ssDNA 5′-GGA CTA GTC TAG GCG AAC GTT TAA GGC GAT CTC TGT TTA CAA CTC CGA-3′ labeled at 5′ with Alexa 488 and at 3′ with Atto 647N or Alexa 647 (IBA, Göttingen, Germany) with complementary unlabeled ssDNA 5′-TCG GAG TTG TAA ACA GAG ATC GCC TTA AAC GXT CGC CTA GAC TAG TCC-3′ (Eurofins, Ebersberg, Germany). The hybridization details can be found in ref. ^40^. A high degree of dual labeling (aimed to be 100%) was verified by IBA using ESI-TOF mass spectrometry and by own absorption spectroscopy measurements. For multi-point calibration measurements (in addition single labeled dsDNA is needed), we employed accordingly single labeled ssDNA (either with Alexa 488 or with Atto647N).

### Isolation and labeling of ribosomes

For the translation initiation experiments the RNase deficient *E. coli* K-12 strain CAN20/12E (RNase BN^−^, II^−^, D^−^, I^−^)^41^ was used. Ribosomes were isolated by sucrose gradient centrifugation using a zonal rotor as previously described^42^ and re-suspended in Tico buffer [20 mM Hepes-KOH (pH 7.6 at 0 °C), 10 mM magnesium acetate, 30 mM ammonium acetate, 4 mM β-mercaptoethanol]. A reaction with Cy5-NHS-ester functionalized dye (GE Healthcare Life Sciences, Little Chalfont, UK) in labeling buffer [50 mM Hepes-KOH (pH 7.5), 10 mM MgCl_2_, 100 mM KCl] followed for 20 min at 37 °C, using a 20-fold excess of dye to minimize the unlabeled fraction of ribosomes. The excess of dye was removed by pelleting the ribosomes through a 1.1 M sucrose cushion. The concentration of Cy5 and ribosomes was determined spectroscopically in a NanoDrop spectrophotometer (Thermo Fisher Scientific, Waltham, USA) using the absorption coefficients *ε*_*cy5*_ = 2.5 × 10^5^ M^−1^ cm^−1^ (at *λ* = 647 nm) and *ε*_*70S*_ = 4.2 × 10^7^ M^−1^ cm^−1^ (at *λ* = 254 nm) respectively. The label ratio was calculated to be ~6 Cy5 dyes per 70S ribosome. Labeled ribosomes were dissociated into 50S and 30S subunits by sucrose gradient centrifugation in dissociation buffer [20 mM Hepes-KOH (pH 7.6 at 0 °C), 0.97 mM magnesium acetate, 200 mM ammonium acetate, 4 mM β-mercaptoethanol]. The concentration of ribosomal subunits was determined again spectroscopically using the absorption coefficients *ε*_*cy5*_ (see above), *ε*_*50S*_ = 2.8 × 10^7^ M^−1^ cm^−1^ and *ε*_*30S*_ = 1.4 × 10^7^ M^−1^ cm^−1^ (the latter two at *λ* = 254 nm), respectively. The label ratio for the isolated subunits were calculated to be ~3 Cy5 dyes per 50S and per 30S subunit, each. Finally, the labeled subunits were re-associated with unlabeled counterparts to form empty 70S ribosomes, labeled only on the 50S or only on the 30S subunit. The re-association reaction was performed at 40 °C for 1 h using a 50% excess of 30S with respect to 50S^43^. For analyzing the long term stability and the structural integrity of the 70S complex we also re-associated Cy5-labeled 50S with Alexa488-labeled 30S subunits to form double-labeled 70S ribosomes. Subsequently stability and integrity of labeled 70S ribosomes was validated by BTCCD measurements (Supplementary Fig. 5).

### DNA construct for cell-free protein synthesis (CFPS)

The DNA construct expressing the GFP variant Emerald (GFPem) together with the C-terminal SecMstr AP was described previously^11^.

### CFPS reactions

The PURE system^44^ without ribosomes (PURExpress Δ ribosomes, NEB #E3313, New England Biolabs, Ipswich, USA) was used in all experiments. Reactions were performed according to the manufacturer’s protocol containing 500 nM of labeled ribosomes and 5.5 nM of linear DNA construct. Additionally 5 nM of anti-sense tmRNA (AS-tmRNA)^45^, complementary to the tmRNA sequence was added to suppress tmRNA-induced ribosome-nascent chain dissociation^46,47^. For the translation initiation +50S reaction 2.5, 7.5 and 10 μM of unlabeled 50S subunits were added to the reaction mix, which results in a 5-fold, 15 fold and 20-fold excess of unlabeled 50S. After completion of the synthesis reactions the diffusion properties were checked by fluorescence correlation spectroscopy and subsequently the solutions were diluted to a final concentration of labeled ribosomes in the order of a few picomolar for single-molecule measurements.

### Confocal fluorescence measurements and data acquisition

All experimental details of the performed measurements were described previously, for the DNA samples in refs. ^40,48^, for translation initiation with ribosomes in ref. ^11^ and for FRET-based biosensors (see Supplementary Note 3) in ref. ^38^. Briefly, all measurements were performed with a MicroTime200 (Picoquant, Berlin, Germany) equipped with pulsed laser diodes, an UPLSAPO 60X/1.2NA objective, a 75 µm pinhole and with two/four silicon avalanche photo-diodes detectors. The lasers were operated at a frequency of 20 MHz and individual excitation power intensities were set for the following samples. The 488 nm and 640 nm excitation was used for DNA nano-beads with 4.2 and 3.7 μW, respectively; dsDNA with 21 and 18 μW, respectively; and ribosome/GFP constructs with 21 and 16 μW, respectively. A 440 nm and 510 nm excitation was used for the FRET-biosensors with 20 and 17 μW, respectively. To avoid any by chance coincidences of diffusing molecules the sample concentrations were carefully checked by FCS before each BTCCD measurement. For this purpose, the average number of each dye (color) within the detection volume at a time was adjusted at <*N*> ~ 0.01. Aliquots from stock solutions of all samples employed in this study were diluted down to above mentioned concentrations and were measured for 20 min time intervals. A sequence of several subsequent 20 min measurements were summed up until a few thousand up to a few ten thousand bursts of each detection color were measured (typically 20–60 min data acquisition time for a data set). A more detailed discussion on the total data acquisition time is given in Supplementary Note 9. A typical time trace of a double-labeled sample and how the burst show up in plots which make use of the IPL time are given in Supplementary Fig. 6. The identification of single-molecule bursts is described in detail in ref. ^40^. While for the calibration and the biosensor samples a single sample aliquot was used to obtain a full sample data set, the ribosomes samples were replaced every 30 min by a new sample aliquot. An inspection of crucial measuring parameters (e.g., number of burst per time, brightness of bursts) of all 20 min measuring intervals within a full data set helped to validate the sample stability.

### Two-color coincidence detection analysis

After identification of all bursts in both channels, burst that contain only a single photon are discarded because they would induce an artificially small dwell time and high molecular brightness. The start time of each burst corresponds to the macro time tag of the first photon of that burst and, accordingly, the end time of a burst is defined as the macro time tag of the last photon. The burst duration is defined as the difference between its start and end time. Bursts that last more than 100-fold longer than the average burst duration are also discarded because they might belong to contaminations or aggregates. For all remaining bursts the burst intensity, i.e. the number of photons between the start and end time, and the mean number of photons per burst is calculated. In the coincidence analysis the brightness threshold is normalized to that mean number of photons in a burst and continuously increased. The coincidence is calculated for each channel independently and for each value of the brightness threshold only bursts that have more photons as defined by the brightness threshold are considered for analysis. Subsequently, the coincidence of these bright bursts to all detected bursts in the other channel is calculated. Two bursts are considered as coincident if the start or end time tag of one burst is within the start and end time tags of the other burst. The fraction of coincident bursts is calculated for each brightness threshold (e.g., for red channel *f*_R_ = *N*_RB_/*N*_R_, see Supplementary Fig. 1) as the number of coincident bursts (*N*_RB_) normalized to the number of selected bursts (*N*_*R*_). The related values for all presented measurements are given in the Supplementary Note Tables N1.3-5. Here the error of the fractions of coincidence values is determined by the statistical error σ of the number of bursts as given by Poisson statistics with $\sigma = \sqrt N$ (see also Supplementary Note 2). The data analysis was performed using self-written Matlab routines (Mathworks, Natick, MA, USA). OriginPro (9.0.0 G, 64 bit) was used to produce the graphical presentation of the obtained results.

### Reporting summary

Further information on research design is available in the Nature Research Reporting Summary linked to this article.

## Supplementary information

Supplemental Material

Description of Additional Supplementary Materials

Supplementary Data 1

Supplementary Data 2

Supplementary Data 3

Supplementary Data 4

Supplementary Data 5

Supplementary Data 6

Supplementary Data 7

Supplementary Data 8

Reporting Summary

## Data Availability

Data that support findings of this study are available from the corresponding author upon reasonable request. Original data to produce plots shown in Figs. 1b–d, 2a–c, 3c, d are available as Supplementary data files.

## Code availability

Code in form of MatLab routines have been made freely available via GitHub at https://github.com/BTCCD/BTCCD.
