# Supplementary material for: Brightness-gated two-color coincidence detection unravels two distinct mechanisms in bacterial protein translation initiation
Source: Commun Biol. 2019 Dec 6;2:459. doi: 10.1038/s42003-019-0709-7 (PMC6897966; doi:10.1038/s42003-019-0709-7)
Supplement: Supplementary file 1 — Supplemental Material [file 42003_2019_709_MOESM1_ESM.pdf]

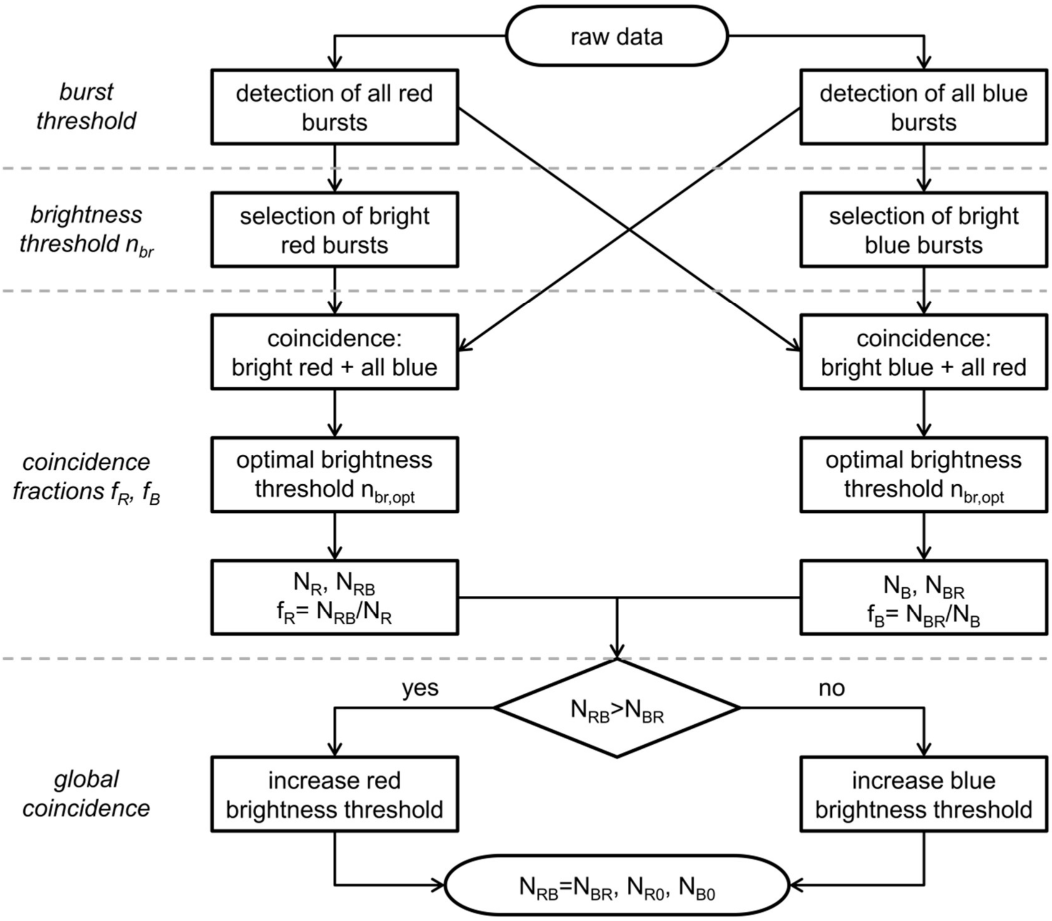

Supplementary Figure 1: Flow chart of brightness-gated two-color coincidence detection. Different thresholds for a proper burst identification and selection are presented. The number of selected burst N_R and N_B and the number of coincident bursts N_{RB} and N_{BR} define the coincidence fractions f_R and f_B of the red and blue channel, respectively. The global coincidence analysis quantifies the final fractions of the three essential species (N_{R0}: only red labeled, N_{B0}: only blue labeled, $N_{RB} = N_{RB}$ red and blue labeled) which are present in the ensemble of a sample.

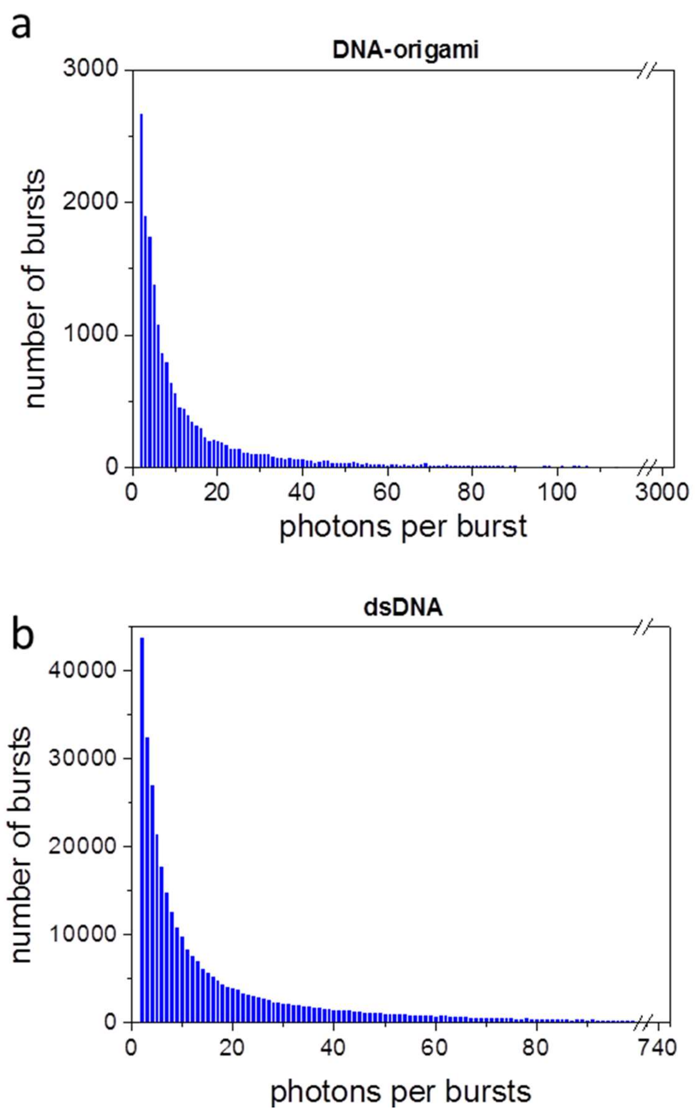

Supplementary Figure 2: Distribution of photons per burst in the blue channel (Alexa 488) for (a) multiple labeled DNA-origami nano-beads and (b) single-labeled dsDNA.

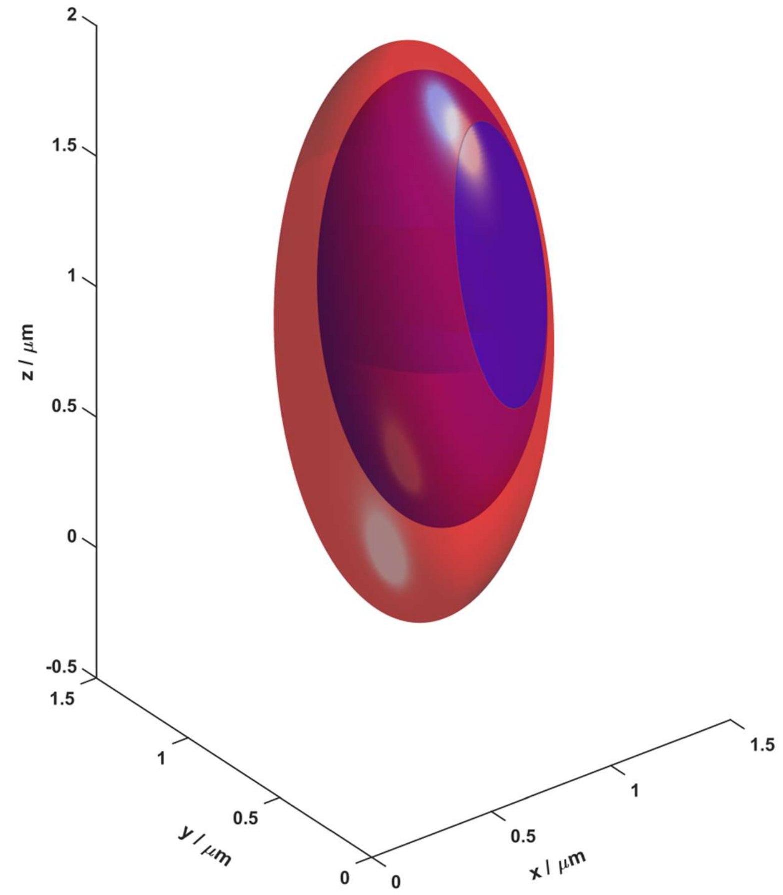

Supplementary Figure 3: Example of a typical raster scanning image performed with the confocal microscope to determine blue and red detection volumes using 0.1µm TetraSpeck beads. Image is reconstructed from x-y, x-z and y-z scans. Here the light red represents a volume of exclusive red detection, the blue color a volume of exclusive blue detection, and the dark red color a volume of blue and red detection.

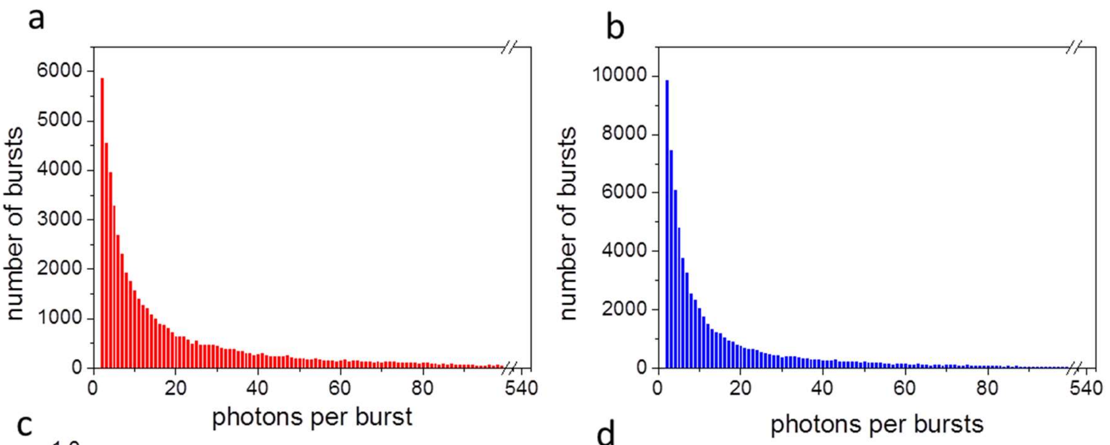

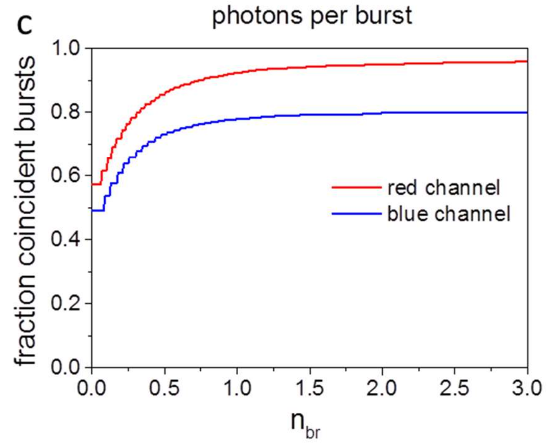

d

N_{BR}, N_{RB}	N_{R0}	N_{B0}	N_{tot}
10,500	500	2,800	13,800
76 %	4 %	20 %	100 %

Supplementary Figure 4: BTCCD analysis of single dye pair dsDNA labeled with Alexa 488 and Alexa 647. (a) Histogram of number of photons per burst for red channel (Alexa 647). (b) Histogram of number of photons per burst for blue channel (Alexa 488). (c) Fraction of coincident bursts shown as a function of normalized brightness threshold n_{br}. Coincidence fractions level off at 0.95±0.01 and 0.79±0.03 for the red and blue channel, respectively. (d) Absolute number of bursts and corresponding statistical weights as obtained by global coincidence analysis.

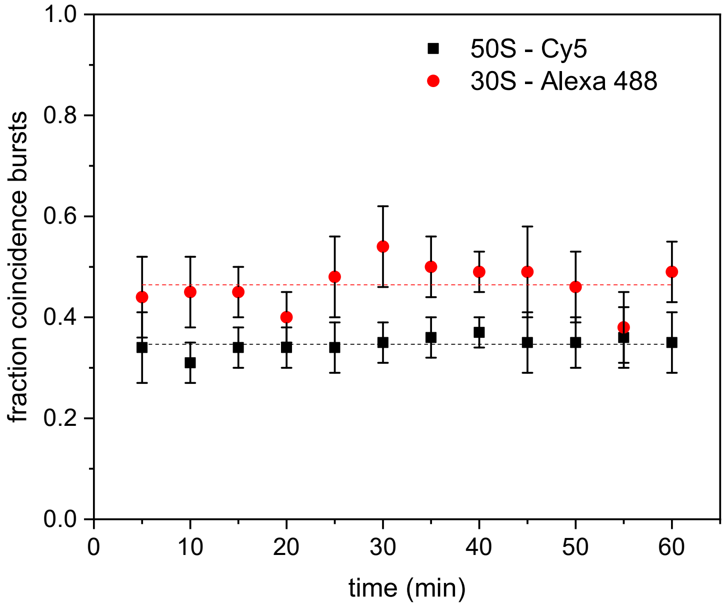

Supplementary Figure 5: Stability of 70S ribosomes at low concentrations (~pM) is investigated with Cy5 labeled 50S subunits and Alexa488 labeled 30S subunits. Consecutive 5 minutes coincidence measurements are performed over one hour. Experimental errors are determined by the burst statistics (see Methods). Coincidence fractions are stable for one hour (fluctuating around dashed lines) and, hence, no dissociation of 70S complex due to the low concentration is occurring during that time window.

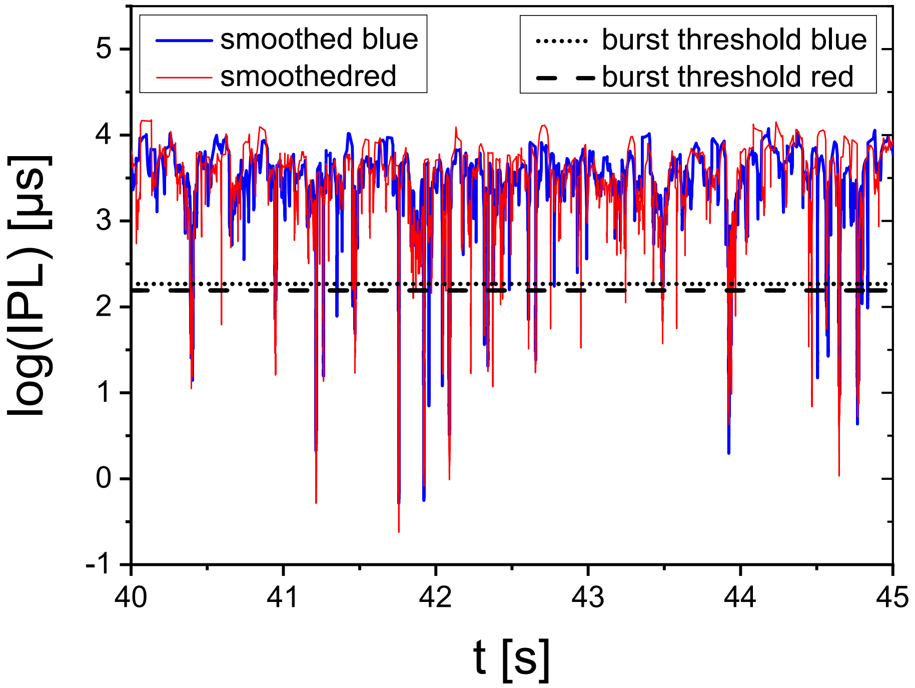

Supplementary Figure 6: A typical time trace of single molecule bursts of nano-bead sample for two different colors is shown for the inter-photon lag time.

Supplementary Note 1: Impact of burst detection parameters on BTCCD results

The impact of the burst detection parameters on the coincidence results was tested by varying the parameters and performing each time a new BTCCD analysis. First, solely the smoothing values of the inter-photon lag (IPL) time traces or solely the IPL burst thresholds were varied simultaneously for both channels. Second, the smoothing values or the burst thresholds were varied conversely, i.e. it was increased for one channel and decreased for the other channel or vice versa. The coincidence fractions as a function of brightness threshold are shown for the single-labeled dsDNA (Alexa 488/Atto 647N) in Figure N1.1 for varying the IPL smoothing value, in Figure N1.2 for varying the IPL burst threshold, in Figure N1.3 for conversely changing the smoothing value, and in Figure N1.4 for conversely changing the burst threshold. Here, the brightness thresholds are plotted on an absolute scale because the burst detection parameters have a pronounced impact on the average burst brightness (see Suppl. Tab. N1.1) and, hence, using the normalized brightness thresholds would distort the comparison.

In the case of a simultaneous change of the parameters (see Figs. N1.1 and N1.2) the coincidence fraction of red bursts as a function of brightness threshold shows some variance, whereas that of blue bursts is almost unaffected. We interpret this as a higher robustness of the red bursts against a variation of the burst detection parameters. Most important, all coincidence fractions saturate at approximately the same value.

A converse change of the burst detection parameters (see Figs. N1.3 and N1.4) has the strongest impact on the coincidence fractions which reveals in different shapes of the coincidence fractions as a function of brightness threshold. For example, using a low IPL threshold for the red channel and a high IPL threshold for the blue channel leads to a shift towards higher red coincidence fractions and a faster saturation in comparison to the original thresholds. In contrast, the blue coincidence fraction starts at lower values and saturates at higher brightness thresholds. This can be understood as a preselection of bright red bursts and a detection of dim bursts in the blue channel. Again, the saturation values of the coincidence fractions are not remarkably affected by the burst detection parameters.

The burst detection parameters and the obtained coincidence values are summarized in Supplementary Table N1.1-6. Although the burst detection parameters are varied beyond a reasonable range, the coincidence fractions are remarkably stable.

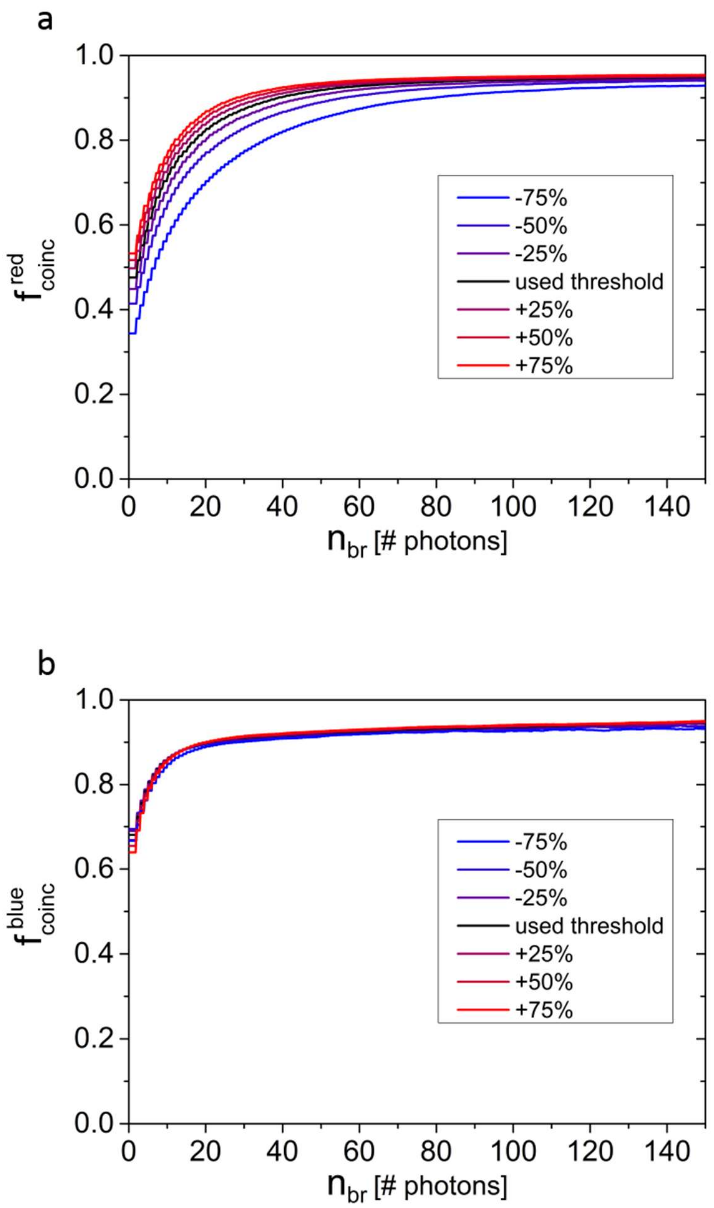

Supplementary Figure N1.1: Fraction of coincident red (a) and blue (b) bursts as a function of brightness threshold for single-labeled dsDNA (Alexa 488/Atto 647N). The IPL threshold is varied simultaneously for both channels from the used threshold (black lines) by the relative changes given in the legend. Note, that smaller IPL thresholds correspond to bursts with higher photon counts. The red coincidence fraction shows generally a stronger dependency on the IPL thresholds, however, all coincidence fractions saturate at approximately the same value.

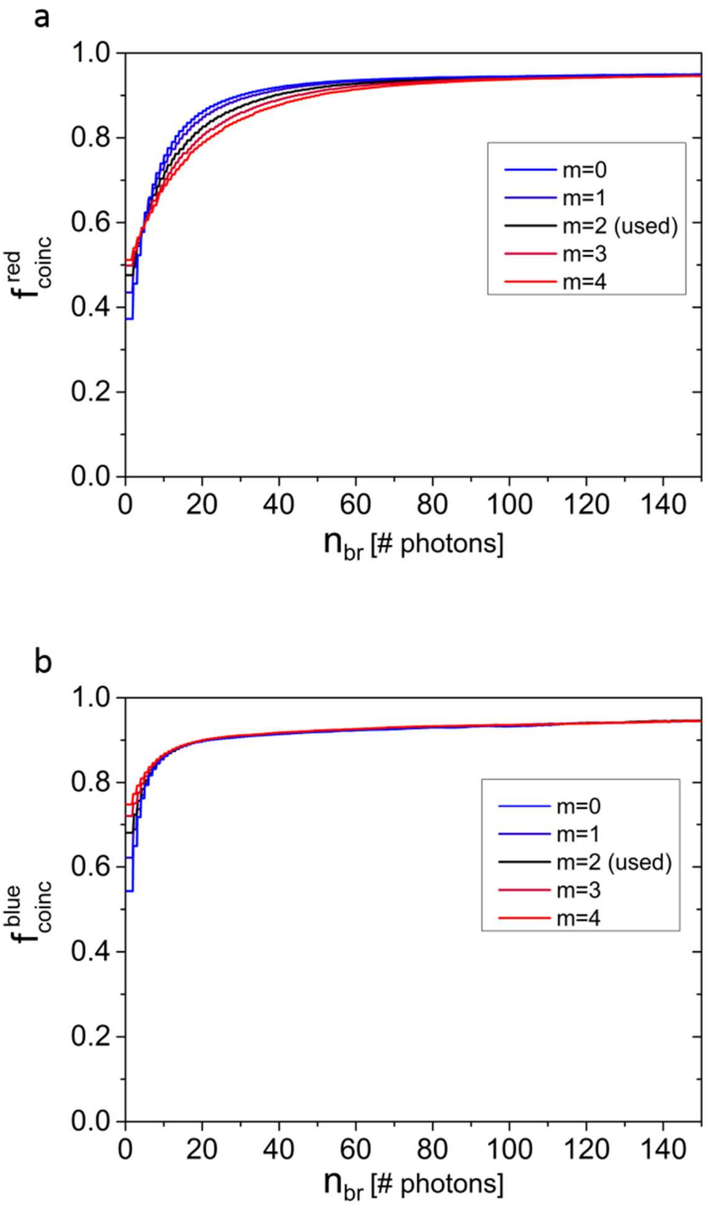

Supplementary Figure N1.2: Fraction of coincident red (a) and blue (b) bursts as a function of brightness threshold for single-labeled dsDNA (Alexa 488/Atto 647N). The IPL smoothing parameter m is varied simultaneously for both channels around the used value (black lines). Note, that large smoothing values will filter out dim bursts. The red coincidence fraction shows generally a stronger dependency on the smoothing parameter, however, all coincidence fractions saturate at approximately the same value.

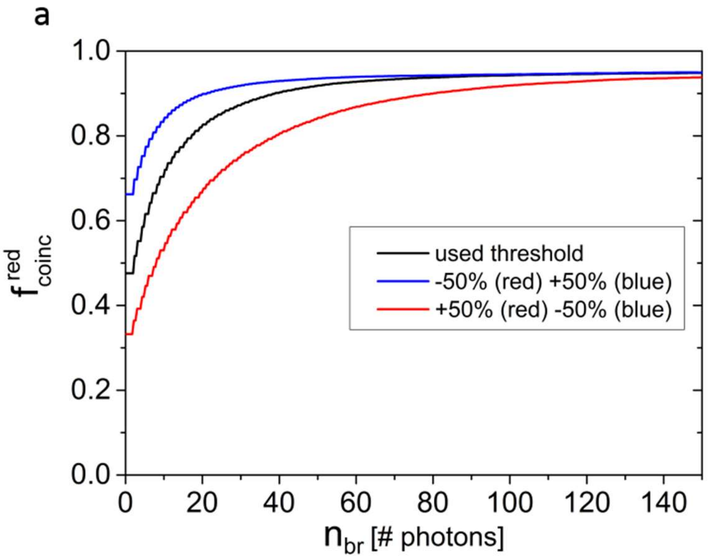

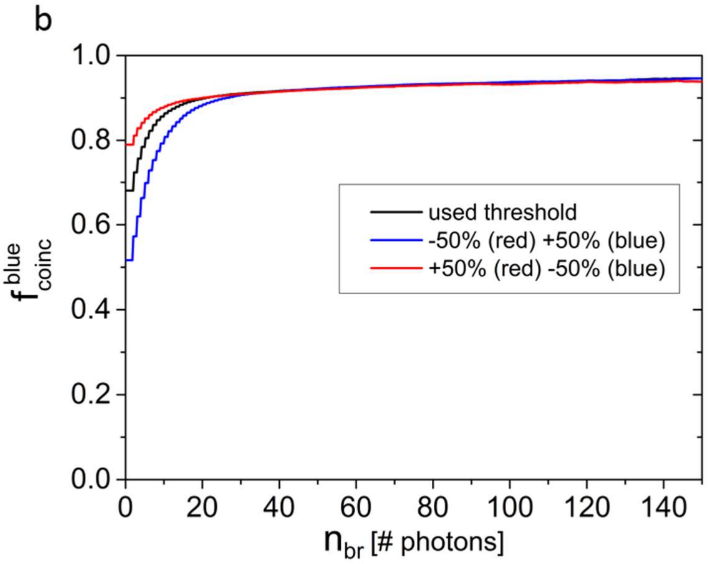

Supplementary Figure N1.3: Fraction of coincident red (a) and blue (b) bursts as a function of brightness threshold for single-labeled dsDNA (Alexa 488/Atto 647N). The IPL threshold is varied from the used threshold (black lines) conversely for both channels by a relative change of +50%/-50% or vice versa. The red and blue coincidence fractions show an opposite dependency on the threshold combinations because a reduction of the IPL thresholds leads to a preselection of bright bursts in the respective channel and, hence, to higher coincidence fraction and a saturation at smaller brightness thresholds.

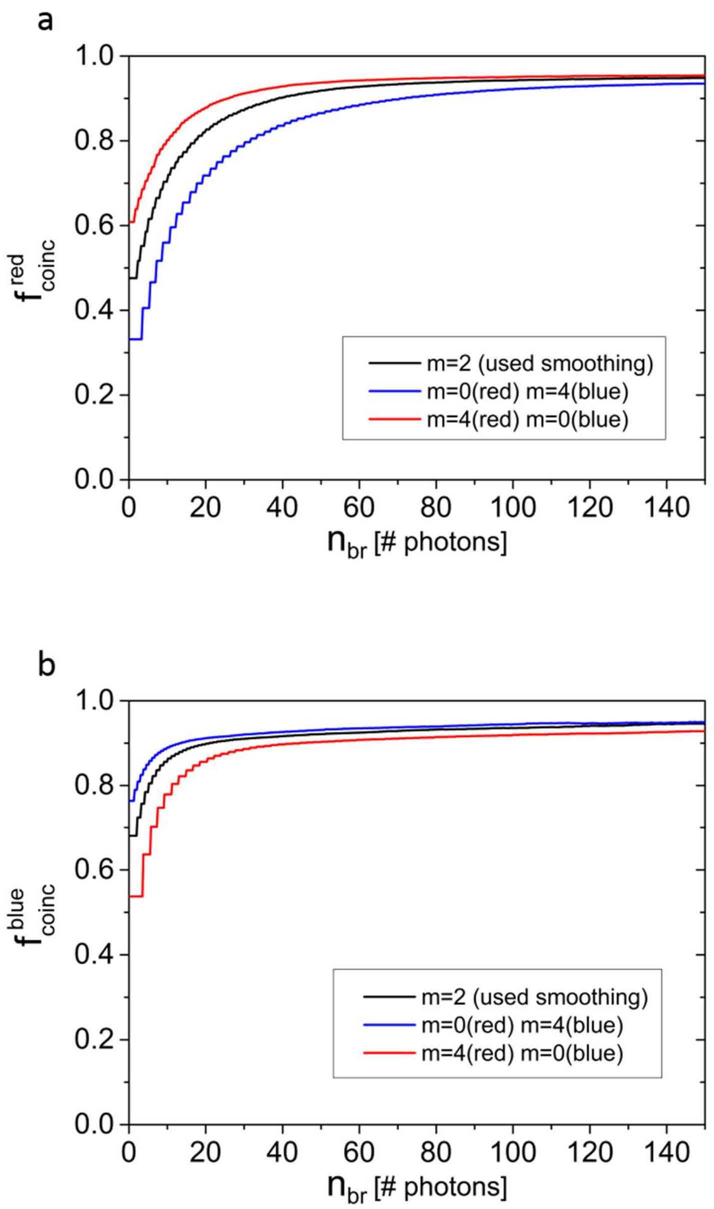

Supplementary Figure N1.4: Fraction of coincident red (a) and blue (b) bursts as a function of brightness threshold for single-labeled dsDNA (Alexa 488/Atto 647N). The IPL smoothing parameter m is varied from the used value (black lines) conversely for both channels. The red coincidence fraction shows generally a stronger dependency on the changes of the IPL smoothing parameters, however, all coincidence fractions saturate at approximately the same value.

IPL burst thresholds IPL_R/IPL_B	average brightness of red burst [photons]	average brightness of blue burst [photons]	optimal red brightness threshold [photons]	optimal blue brightness threshold [photons]	fraction selected red burst	fraction selected blue bursts	red coincidence fraction $f_{coinc,R}$	blue coincidence fraction $f_{coinc,B}$
110µs / 120µs	39.4	24.7	114	109	9.4%	4.5%	0.945±0.007	0.937±0.011
110µs / 120µs	18.7	33.9	94.1	117	4.6%	6.3%	0.937±0.006	0.943±0.011
110µs / 120µs	53.2	11.9	116	76.6	13.3%	2.6%	0.953±0.006	0.913±0.009
55µs / 180µs	32.8	25.4	59.7	127	15.9%	3.7%	0.939±0.005	0.941±0.011
165µs / 60µs	43.2	22.1	155	70.9	6.9%	7.4%	0.939±0.008	0.928±0.009

Supplementary Table N1.1: Robustness of coincidence fractions measured for the dsDNA (Alexa 488/Atto 647N) for varied burst detection parameters. First line shows the initial parameters. Relative changes of ±100% of the inter-photon lag (IPL) smoothing value m and ±50% of the IPL threshold value are applied conversely for the red and blue channel. While most burst quantities change remarkably, the coincidence fractions are extremely robust. Almost all coincidence fractions agree within the errors.

IPL smoothing values m_R / m_B	IPL burst thresholds IPL_R/IPL_B	average dwell time of red burst [ms]	average dwell time of blue burst [ms]	average molecular brightness of red bursts [kHz/molecule]	average molecular brightness of blue bursts [kHz/molecule]
2 / 2	110µs / 120µs	0.945	0.737	35.0	33.5
0 / 4	110µs / 120µs	0.356	1.05	42.4	33.6
4 / 0	110µs / 120µs	1.33	0.282	35.9	40.2
2 / 2	55µs / 180µs	0.514	1.03	54.0	25.2
2 / 2	165µs / 60µs	1.34	0.405	27.5	53.4

Supplementary Table N1.2: Dependency of burst parameters of dsDNA (Alexa 488/Atto 647N) on varied burst detection parameters. First line shows the initial parameters.

sample	IPL smoothing values m_R / m_B	IPL burst thresholds IPL_R/IPL_B [μs]	background IPD red channel [ms]	background IPD blue channel [ms]	average dwell time red burst [ms]	average dwell time blue burst [ms]	average molecular brightness red bursts [kHz/molecule]	average molecular brightness blue bursts [kHz/molecule]
nano-bead	2 / 2	185 / 155	3.16	3.55	2.47	2.14	22.8	21.5
dsDNA Al488/At647N	2 / 2	110 / 120	2.00	1.12	0.945	0.737	35.0	33.5
dsDNA Al488/Al647	2 / 2	125 / 110	3.16	1.00	0.918	0.669	31.0	35.1
ribosome # 1a (ref)	2/2	300/600	2.70	2.50	2.49	2.10	15.1	8.1
ribosome # 1b	2/2	300/600	2.80	2.49	2.27	2.00	17.4	13.4
ribosome # 2a (ref)	2/2	300/600	2.75	2.45	3.39	2.50	13.4	9.3
ribosome # 2b	2/2	300/600	2.80	2.50	2.96	2.51	12.4	8.6
ribosome # 2c	2/2	300/600	2.65	2.45	3.61	2.62	10.8	6.5
ribosome # 2d	2/2	300/600	2.90	2.50	4.59	2.52	5.9	9.0
ribosome # 3a (ref)	2/2	300/600	2.70	2.55	3.10	1.86	12.8	10.8
ribosome # 3b	2/2	300/600	2.65	2.55	2.62	1.47	14.3	10.6
ribosome # 4a (ref)	2/2	300/600	2.60	2.70	2.88	1.30	12.5	9.7
ribosome # 4b	2/2	300/600	2.60	2.70	2.93	1.33	12.8	9.6
ribosome # 5a (ref)	2/2	300/600	2.65	2.45	3.35	2.07	12.6	11.2
ribosome # 5b	2/2	300/600	2.60	2.40	2.99	1.31	13.6	10.9

Supplementary Table N1.3: Burst parameter of employed samples.

sample	average brightness of red burst [photons]	average brightness of blue burst [photons]	optimal red brightness threshold [photons]	optimal blue brightness threshold [photons]	fraction selected red burst	fraction selected blue bursts	red coincidence fraction $f_{coinc,R}$	blue coincidence fraction $f_{coinc,B}$
nano-bead	121	124	58.1	44.6	13.9%	18.4%	0.977±0.022	0.976±0.022
dsDNA Al488/At647N	39.4	24.7	114	109	9.4%	4.5%	0.945±0.007	0.937±0.011
dsDNA Al488/Al647	30.1	23.1	57.8	155	13.3%	2.6%	0.950±0.014	0.818±0.033
ribosome # 1a (ref)	57.7	14.8	339	21.0	5.6%	11.5%	0.35±0.03	0.63±0.06
ribosome # 1b	61.2	15.6	276	25	5.4%	14.2%	0.09±0.01	0.18±0.03
ribosome # 2a (ref)	78.9	18.4	306	40.8	6.2%	12.8%	0.25±0.03	0.77±0.12
ribosome # 2b	64.2	11.3	257	22.2	5.8%	9.3%	0.08±0.01	0.23±0.05
ribosome # 2c	36.0	13.1	69.8	19.8	10.9%	35.7%	0.03±0.01	0.14±0.04
ribosome # 2d	57.0	18.5	248	20.0	5.5%	22.7%	0.06±0.02	0.15±0.03
ribosome # 3a (ref)	62.4	15.0	262	51	5.9%	5.6%	0.33±0.02	0.51±0.03
ribosome # 3b	48.3	9.1	217	27.9	5.3%	6.0%	0.15±0.02	0.13±0.02
ribosome # 4a (ref)	58.5	6.98	253	29.9	5.3%	3.1%	0.34±0.02	0.53±0.03
ribosome # 4b	56.4	7.64	255	27.0	5.6%	4.2%	0.08±0.01	0.06±0.01
ribosome # 5a (ref)	72.0	15.8	289	48.9	5.8%	7.9%	0.42±0.02	0.56±0.02
ribosome # 5b	59.3	8.2	232	20.9	6.1%	6.2%	0.06±0.01	0.10±0.01

Supplementary Table N1.4: BTCCD parameter of employed samples.

sample	total measurement time [sec.]	number of detected red bursts	Number of detected blue bursts
nano-bead	7200	28,848	21,128
dsDNA Al488/At647N	27600	466,437	362,830
dsDNA Al488/Al647	36000	60,422	75,792
ribosome # 1a (ref)	7200	9261	1948
ribosome # 1b	7200	17064	3777
ribosome # 2a (ref)	3600	10686	1188
ribosome # 2b	3600	9148	1012
ribosome # 2c	3600	16432	890
ribosome # 2d	3600	16973	1863
ribosome # 3a (ref)	14400	51139	25622
ribosome # 3b	14400	21332	20465
ribosome # 4a (ref)	14400	42585	36862
ribosome # 4b	14400	17195	15448
ribosome # 5a (ref)	12600	39878	14961
ribosome # 5b	12600	13060	8656

Supplementary Table N1.5: Experimental parameter of employed sample.

Supplementary Note 2: Independent repetitive BTCCD measurements

The reliability of our method is demonstrated with three repetitive measurements performed with three individual samples (100% double labelled dsDNA, see Fig. 2b and Suppl. Fig. N2a,b). As shown in the Tab. N2.2 the obtained coincidence values agree very well across the samples for both channels.

a

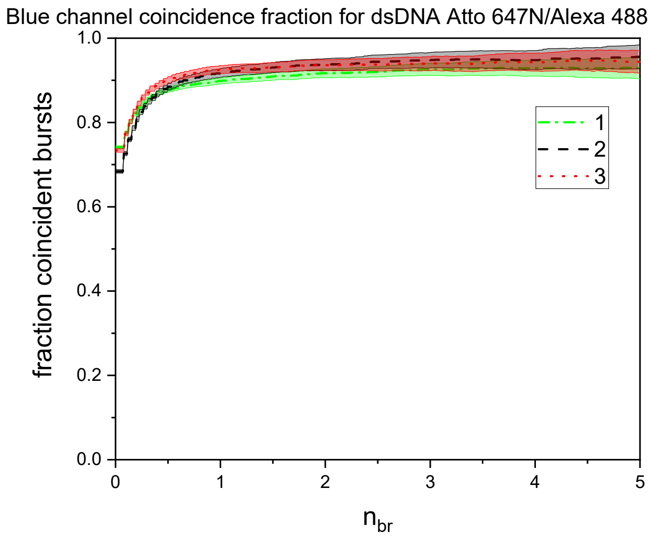

b

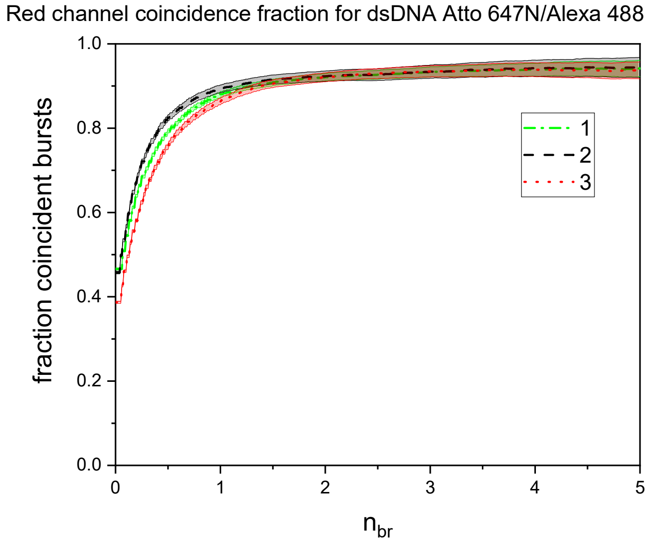

Supplementary Figure N2: Fractions of coincidence bursts plotted as a functions of n_{br}. The results from three independent samples (100% double labelled dsDNA, colored lines) yield within the limits of error the same results. The shaded area around the experimental lines represent the statistical error. Data for sample #2 is identical to that shown in Fig. 2b in the main text of the manuscript.

sample	IPL smoothin g values m_R / m_B	IPL burst thresholds IPL_R/IPL_B [µs]	background IPD red channel [ms]	background IPD blue channel [ms]	average dwell time red burst [ms]	average dwell time blue burst [ms]	average molecular brightness red bursts [kHz/molecule]	average molecular brightness blue bursts [kHz/molecule]
#1: dsDNA Al488/At647N 1	2/2	110/105	2.85	3.14	0.917	0.691	31.3	33.6
#2: dsDNA Al488/At647N 2	2/2	100/100	3.15	3.01	0.939	0.668	37.8	37.6
#3: dsDNA Al488/At647N 3	2/2	110/100	3.05	2.95	0.963	0.647	32.8	36

Supplementary Table N2.1: Burst parameter for dsDNA (Alexa 488/Atto 647N) repetitive measurements.

sample	average brightness of red burst [photons]	average brightness of blue burst [photons]	optimal red brightness threshold [photons]	optimal blue brightness threshold [photons]	fraction selected red burst	fraction selected blue bursts	red coincidence fraction $f_{coinc,R}$	blue coincidence fraction $f_{coinc,B}$
#1: dsDNA Al488/At647N 1	58.1	44.6	121	124	13.9%	18.4%	0.92±0.03	0.91±0.01
#2: dsDNA Al488/At647N 2	39.4	24.7	114	109	9.4%	4.5%	0.945±0.007	0.94±0.01
#3: dsDNA Al488/At647N 3	30.1	23.1	57.8	155	13.3%	2.6%	0.95±0.01	0.93±0.02

Supplementary Table N2.2: Burst parameter for dsDNA (Alexa 488/Atto 647N) repetitive measurements.

sample	total measurement time [sec.]	number of detected red bursts	number of detected blue bursts
#1: dsDNA Al488/At647N 1	3600	156479	115874
#2: dsDNA Al488/At647N 2	3600	82470	64294
#3: dsDNA Al488/At647N 3	3600	115745	77322

Supplementary Table N2.3: Burst parameter for dsDNA (Alexa 488/Atto 647N) repetitive measurements.

Supplementary Note 3: Multi-point calibrations

The reliability of our BTCCD approach was validated by a determination of coincidence values obtained from sample mixtures of single and double labeled species, prepared with well-defined mixing ratios. For this purpose, first a solution of a targeted 100% double labelled dsDNA was measured (see sample in Fig. 2b). This sample shows a very high coincidence (0.91±0.01 for the blue channel and 0.90±0.01 for the red channel, see Tab. N3.1). After this measurements, various mixtures of double labelled dsDNA (BR) and single blue labelled dsDNA (B0) were prepared (mixing ratios of 25%, 50% and 75% fraction of single labelled species) and measured. Finally, a sample with 50% single blue labelled (B0) and with 50% single red labelled (R0) was prepared. Since this sample consist only of single labeled molecules it has to show coincidence values close to zero. Furthermore, the mixture of single labeled molecules with two different colors has to be measured at a sufficiently low molecule concentration in order to avoid chance coincidence. Both requirements are fulfilled, verified by the fact that we obtained very low coincidence values (0.005±0.001 for the red channel and 0.004±0.001 for the blue channel, see Tab. N3.1). Data measured with these samples is shown in panel (a) of Figure N3. In a similar manner measurements were performed with single red labelled dsDNA (R0), as shown in panel (b) of Fig. N3. The targeted mixing ratio (values above the individual curves) agree very good within the limits of error with those obtained from the measurements (see normalized coincidence values in Tab. N3.1). Statistical details of all involved measurements are given in Tab. N3.2-4. In summary, this multi-point calibration demonstrates the reliability of our approach for the determination of all possible coincidence values between 1 and 0.

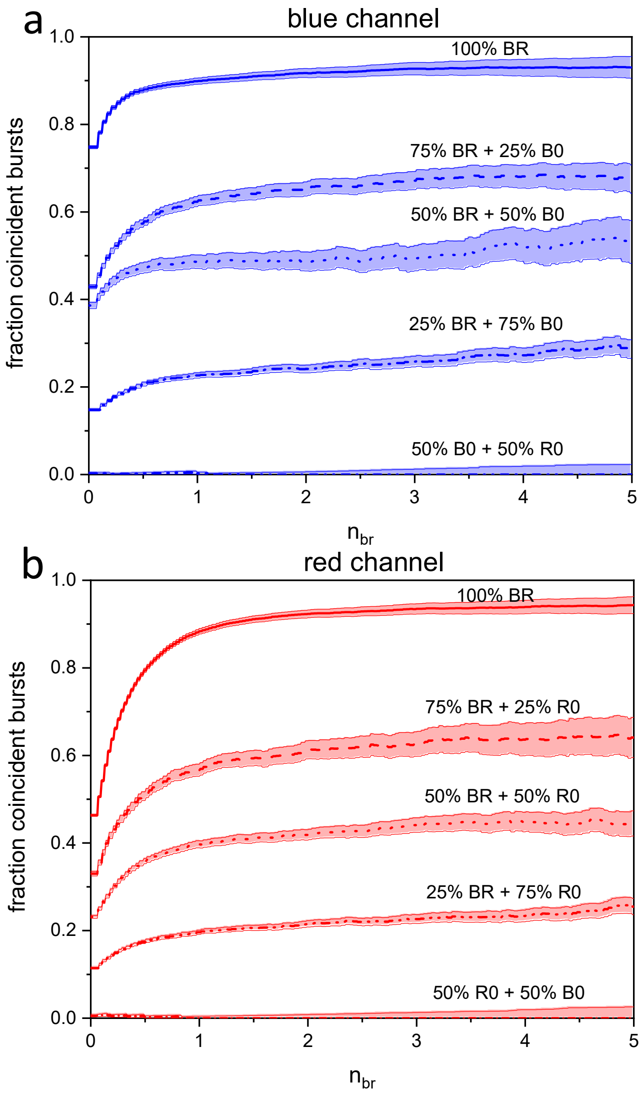

Supplementary Figure N3: Fractions of coincidence bursts are plotted as a function of normalized brightness threshold n_{br}. Results of measurements from sample mixtures consisting of double labelled and single labelled dsDNA molecules are shown here. In two cases samples with a 100% fraction of double labelled molecules (BR) were then mixed with an increasing fraction of single labelled dsDNA, either with single blue labelled molecules (B0) as shown in (a), or with red labelled molecules (R0), shown in (b). The target mixing ratios of the individual components are given above the related curve, while the experimentally obtained coincidence values are given in Tab. N3.1. The shaded area around the experimental lines represent the statistical error.

Targeted mixture	expected coincidence red channel	expected coincidence blue channel	absolute coincidence red channel	absolute coincidence blue channel	normalized coincidence red channel	normalized coincidence blue channel
100% BR	1.00	1.00	0.90±0.01	0.91±0.01	1.00±0.02	1.00±0.02
75% BR + 25% R0	0.75	1.00	0.63±0.03	0.90±0.05	0.70±0.07	1.00±0.07
50% BR + 50% R0	0.50	1.00	0.44±0.02	0.91±0.04	0.49±0.05	1.00±0.05
25% BR + 75% R0	0.25	1.00	0.22±0.01	0.91±0.07	0.24±0.02	1.00±0.06
75% BR + 25% B0	1.00	0.75	0.91±0.03	0.67±0.01	1.00±0.04	0.74±0.04
50% BR + 50% B0	1.00	0.50	0.93±0.03	0.49±0.07	1.00±0.04	0.54±0.09
25% BR + 75% B0	1.00	0.25	0.89±0.03	0.25±0.05	1.00±0.05	0.27±0.06
50% B0 + 50% R0	0.00	0.00	0.005±0.001	0.003±0.001	0.01 ±0.005	0.00±0.01

Supplementary Table N3.1: Coincidence parameters for multipoint calibration measurements. Normalized coincidence values were obtained by the normalizing all values from samples mixtures to the value obtain by the 100% BR sample. The accuracy of our approach is given by the agreement of expected values and measured normalized coincidence values (compare values given in the colored boxes).

sample	IPL smoothing values	IPL burst thresholds IPL_R/IPL_B [µs]	background IPD red channel [ms]	background IPD blue channel [ms]	average dwell time red burst [ms]	average dwell time blue burst [ms]	average molecular brightness red bursts [kHz/molecule]	average molecular brightness blue bursts [kHz/molecule]
	m_R / m_B							
BR dsDNA	2/2	120 / 120	2.85	2.95	0.991	0.785	29.5	30.5
0.5BR+0.5R0	2/2	120 / 120	3.10	2.90	0.903	0.601	29.5	28
0.75BR+0.25R0	2/2	120 / 120	3.39	2.94	0.876	0.562	31.4	28.9
0.25BR+0.75R0	2/2	120/120	3.05	3.15	0.863	0.61	30.1	29.5
0.5BR+0.5B0	2/2	120/120	2.90	2.60	1.069	0.816	34.3	34.3
0.75BR+0.25B0	2/2	120/120	2.95	2.85	1.070	0.757	32.3	34.4
0.25BR+0.75B0	2/2	120/120	3.80	3.10	0.913	0.642	30.7	29.5
0.5 R0+0.5B0	2/2	120/120	2.90	2.55	1.120	0.523	34.4	32.01

Supplementary Table N3.2: Burst parameter for multipoint calibration measurement.

sample	average brightness of red burst [photons]	average brightness of blue burst [photons]	optimal red brightness threshold [photons]	optimal blue brightness threshold [photons]	fraction selected red burst	fraction selected blue bursts
BR dsDNA	33.8	24.7	94.7	64.1	9.37%	12.70%
0.5BR+0.5R0	29.0	16.1	113	70	3.42%	4.19%
0.75BR+0.25R0	29.9	15.3	91.9	70.9	7.48%	3.91%
0.25BR+0.75R0	28.3	17.4	105	87.9	5.56%	3.19%
0.5BR+0.5B0	36.1	19.4	118	86.1	6.01%	7.41%
0.75BR+0.25B0	33.9	16.5	130	70.7	9.33%	16.8%
0.25BR+0.75B0	31.2	18.7	242	160	26.22%	0.58%
0.5R0+0.5B0	38.5	11.2	86.8	36.0	8.11%	3.90%

Supplementary Table N3.3: Burst parameter for multipoint calibration measurement.

sample	total measurement time [sec.]	number of detected red bursts	number of detected blue bursts
BR dsDNA	3600	156,266	116,964
0.5BR+0.5R0	1200	31,781	19,682
0.75BR+0.25R0	1200	17,315	18,610
0.25BR+0.75R0	1200	39,156	11,578
0.5BR+0.5B0	1200	7,795	11,213
0.75BR+0.25B0	1200	26,665	32,466
0.25BR+0.75B0	2400	8,472	29,032
0.5R0+0.5B0	1200	3204	5160

Supplementary Table N3.4: Burst parameter for multipoint calibration measurement.

Supplementary Note 4: Impact of photo-stability and excitation power

When a dual-labeled molecule (e.g. blue and red labeled) transverses the confocal volume there is a certain probability that one of the two fluorophores photo-bleaches, a fact that would bias the coincidence results. Our BTCCD approach can reduce the impact of photo-bleaching on the coincidence fractions to some extent. The probability that a fluorophore bleaches immediately after entering the confocal volume is low. In contrast, if bleaching occurs, it is likely to happen after the molecule traversed already some part of the confocal volume. It is thus also likely that it already emitted enough photons to be recognized by the burst selection threshold. Since our BTCCD compares bright bursts in one channel with all burst in the other channel, the coincidence is still detected although one fluorophore bleached. However, this bleached molecule could reoccur in the confocal volume[1] now as physically single-labeled and could be identified incorrectly. Assuming that both dyes have a similar photo-stability there is an increased probability that the remaining dye will also bleach and, hence, the burst will not enter the analysis because it is not bright. The impact of photo-stability of a dye can be investigated by varying the excitation intensity of the corresponding excitation laser. However, this will also affect the effective size and overlap of the confocal volumes. We therefore varied the laser powers simultaneously for both lasers. For the case of less photo-stable dyes with relatively long-lived cis-trans isomerization states (e.g., Alexa 647, Cy5) we observe an impact of the laser power on coincidence fractions, but this impact seems to be rather small in the regime of low excitation powers (Supplementary Fig. N4a). The effect of changing only one excitation intensity and keeping the other one constant is shown in Supplementary Figure N4b. A difference of the coincidence fraction due to differences in the confocal overlap can be observed for low brightness threshold. This effect levels off for larger brightness threshold which proves the robustness of BTCCD.

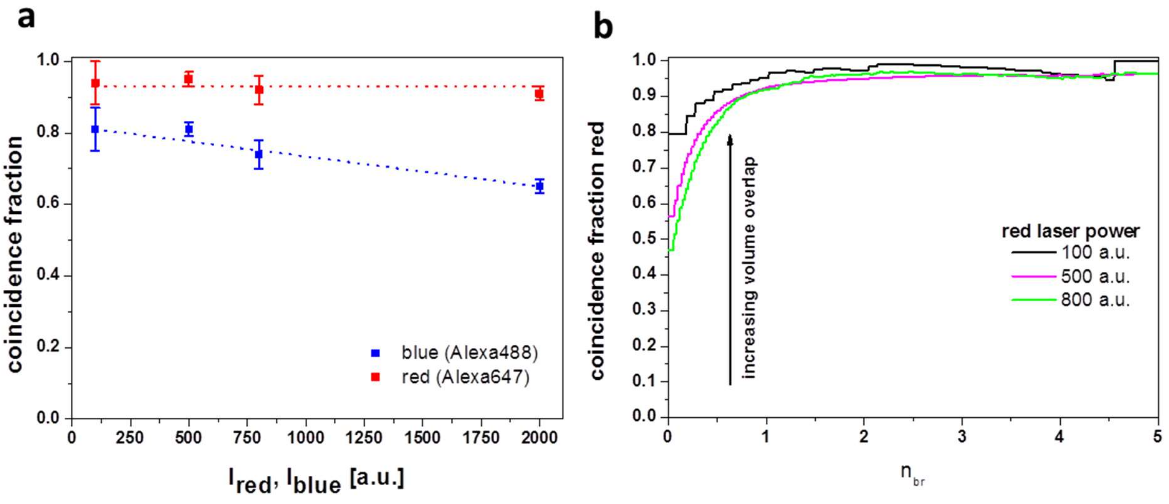

Supplementary Figure N4: (a) Photo-stability of dsDNA (Alexa 488/Alexa 647) reference. Blue and red coincidence fractions are shown as a function of red and blue laser power. The red coincidence fractions remain constant over the range of laser powers (red dotted line) indicating that the blue dye (Alexa 488) is not bleaching. The blue coincidence however shows a linear decreasing trend for increasing laser powers (indicated by blue dotted line) which is due to bleaching or long-lived dark states of the red dye (Alexa 647). The results shown in Supplementary Figure 4 were obtained using 500 a.u. for both lasers so that the photo-stability of Alexa 647 has only a minor effect. (b) Decreasing the red laser power increases the volume overlap. Red coincidence fractions of same sample as in (a) are plotted as a function of brightness threshold for various red laser powers. The lower the laser power is, the higher the starting coincidence fractions ($n_{br} = 0$). The saturating coincidence fraction (above $n_{br} \sim 0.8$) is rather similar for all laser powers, which demonstrates that our BTCCD is independent of the laser power (at moderate laser powers).

Supplementary Note 5: Determination of optimal brightness threshold

The optimal brightness threshold is determined for each channel independently as a trade-off between accuracy and precision of the respective coincidence fraction. Increasing the brightness threshold leads to a higher and, hence, more accurate value of the coincidence fraction as outlined in the main text. However, when the plateau of the coincidence fraction is reached a further increase of the brightness threshold leads only to a slight increase of the coincidence fraction. At the same time, the precision of the determined coincidence fraction decreases because an increase of the brightness threshold leads to less selected bursts that enter the calculation. We define the optimal brightness threshold as the value where the gain of accuracy is overcompensated by a loss in precision. In practice, the optimal brightness threshold is determined by plotting the relative accuracy and the relative precision as a function of brightness threshold and determining the intersection as shown in Supplementary Fig. N5. It is assumed that the true coincidence fraction is reached at the highest brightness threshold. This definition requires a careful setting of the highest brightness threshold. If it is chosen too high, the coincidence fraction is already fluctuating strongly because of the low precision (few bursts).

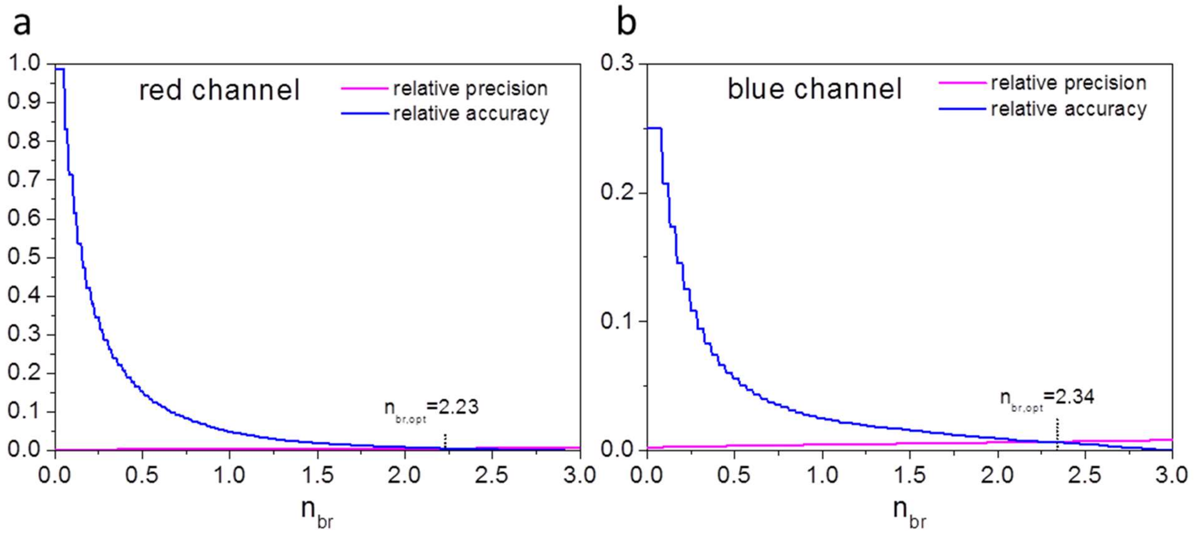

Supplementary Figure N5: Determination of optimal brightness threshold $n_{br,opt}$ for red channel in (a) and blue channel in (b). Data of the single dye dsDNA Alexa 488/Atto 647N reference sample is shown. The optimal brightness threshold is reached when the further increase of accuracy (blue line) for higher brightness threshold is canceled out by a lower precision (pink line) because of the lower number of bursts that enter the analysis. The intersection of both lines also determines the relative error of the coincidence fractions.

Supplementary Note 6: Genetically encoded FRET-based biosensors

We applied our BTCCD analysis to two genetically encoded FRET-based biosensors, for which one of them respond to (i) glucose concentrations[2] and the other to (ii) macromolecular crowding[3]. In both types of sensors, variants of the cyan fluorescent protein (CFP) and the yellow fluorescent proteins (YFP) were employed as the donor and the acceptor, respectively. The sensor for glucose quantification consists of glucose binding protein MglB fused to CFP variant mTurquoise2 as donor and YFP variant Venus as acceptor. The glucose sensor used here is identical to construct no.1 in ref.[2]. The crowding sensor consists of an alpha helical linker with a length of 118 residues fused to mCerulean3 as the donor and mCitrine as the acceptor. The sensor is identical to construct GE in ref.[3].

The coincidence fractions as a function of brightness threshold are shown for both sensors in Supplementary Figure N6a,b. When applying BTCCD to FRET-based biosensors the sum of all photons in the donor and acceptor channel after donor excitation was used as the signal that probes donor presence. First, acceptor photons arise due to FRET from an excited donor and, second, there is a considerable crosstalk of donor photons into the acceptor channel[2]. We do not treat direct acceptor excitation by the laser for donor excitation since it is practically negligible. This analysis scheme is of course applicable to FRET samples in general and was already used for TCCD[4]. For the glucose sensor, the saturation values for the fraction of coincident YFP and CFP bursts are determined to be 82.0 ± 1.1% and 59.0 ± 0.5%, respectively (Supplementary Fig. N6a). Accordingly, the donor-only fraction is 41.0%, which means that the sensitivity of the sensor could be significantly improved by increasing the extent of acceptor maturation. The crowding sensor shows almost full YFP coincidence (96.5±3.3%) and a higher CFP coincidence fraction of 81.2 ± 3.1% as compared to the glucose sensor (Supplementary Fig. N6b). Consequently, we obtain a donor-only fraction for the crowding sensor of only 18.8 %. Supporting these results, global BTCCD analysis shows that glucose sensor production yields only 53% of operable molecules, whereas 80% of all crowding sensor are functional (Supplementary Fig N6c).

We attribute the differences in coincidence to (i) different CFP/YFP variants (mTurquoise2/Venus for the glucose sensor and mCerulean3/mCitrine for the crowding sensor) and (ii) to different complexity in the sensor design. In the glucose sensor the CFP is internally fused to a glucose binding protein which might hamper folding and subsequent fluorophore maturation[5]. In contrast, the sensing domain of the crowding sensor has no tertiary structure[3] which might interfere with

folding and maturation of the FPs and, hence, leads to a better maturation efficiency and higher coincidence ratios.

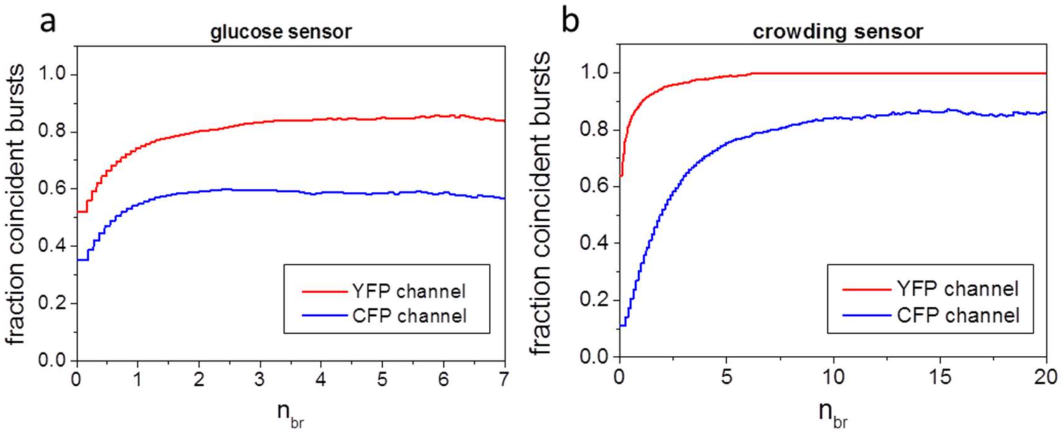

c

biosensor	$N_{CFP+YFP}$	$N_{YFP\text{-}only}$	$N_{CFP\text{-}only}$	N_{tot}
glucose sensor	10600	2400	7100	20100
	53 %	12 %	35 %	100 %
crowding sensor	1230	40	270	1540
	80 %	3 %	18 %	100 %

Supplementary Figure N6: Coincidence analysis of genetically encoded FRET-based biosensors. (a,b) Fractions of coincident bursts are shown as a function of the brightness threshold n_{br} for both sensor types. (c) Global BTCCD yields numbers of FRET sensors carrying both operative FPs ($N_{CFP+YFP}$), only the donor ($N_{CFP\text{-}only}$), or only the acceptor (N_{YFP}-only). Note, that the global fractions of the number of CFP-only molecules $N_{CFP\text{-}only}$ in (c) are not identical with the practically relevant donor-only fractions. The latter do not depend on the acceptor-only fractions which are not visible in a FRET measurement.

Supplementary Note 7: Translation initiation control measurements

In order to validate and quantify the occurrence of 70S initiation we performed six independent measurements (individual synthesis reactions) with different ratios of unlabeled 50S subunits, namely 5 fold, 15 fold and 20 fold excess of unlabeled 50S subunits (with respect to amount of 70S ribosomes (see Figs. N7.1a,b, N7.2a,b, N7.3b and Fig. 3c,d) and one independent measurement with 30S labeling, again with -30S and +30S unlabeled subunits (see Fig. N7.3a). Based on the assumptions and measured parameters presented in our measurement scheme (see Fig. 3) we can calculate the probability for the occurrence of 70S initiation p_{70} and that of the 30S initiation p_{30} with

$$p_{70} = \frac{N_{GR}^{exc}}{N_{GR}^{ref}} \quad \text{and} \quad p_{30} = \frac{\frac{1}{2}\left(N_{G0}^{exc} + N_{R0}^{exc}\right) + N_{GR,reas}^{exc}}{N_{GR}^{ref}} \qquad \text{eq. 7.1}$$

Here N_{G0}^{exc} and N_{R0}^{exc} should be identical (see Fig. 3b). Therefore, we first determine both values and in the following we take the arithmetic mean of the both. Labeled 50S can be present in solution from dissociated 70S ribosomes (30S binding initiation) and mix with the excess of unlabeled 50S. An additional pathway for dual-labeled 70S is hence re-association with this labeled 50S which results in $N_{GR,reas}^{exc}$ that need to be considered explicitly in our calculations. Since we cannot distinguish between the different double labeled species experimentally, the measured fraction of 70S initiation includes the $N_{GR,reas}^{exc}$ contribution with

$$f_{70S} = \frac{N_{GR}^{exc} + N_{GR,reas}^{exc}}{N_{GR}^{ref}} = \frac{N_{GR}^{exc}}{N_{GR}^{ref}} + \frac{N_{GR,reas}^{exc}}{N_{GR}^{ref}} = p_{70} + p_{reas} \qquad \text{eq. 7.2}$$

Now we can relate this experimentally determined parameter to the real probability of 70S initiation occurrence p_{70} to our measured value with

$$p_{reas} = p_{30} \cdot \frac{N_{\text{labeled 50S in solution}}}{N_{\text{all 50S in solution}}} = p_{30} \frac{p_{30} \cdot c_{ribo}^{ref}}{p_{30} \cdot c_{ribo}^{ref} + x \cdot c_{ribo}^{ref}} = \frac{(p_{30})^2}{p_{30} + x} \qquad \text{eq. 7.3}$$

where c_{ribo}^{ref} is the 70S ribosome concentration (which is the same in the reference and in the excess sample) and x is the excess in the case of adding unlabeled 50S subunits. Assuming that $p_{30} = 1-p_{70}$ we obtain

$$f_{70S} = p_{70} + \frac{(1-p_{70})^2}{(1-p_{70}) + x} \qquad \text{eq. 7.4}$$

Based on the results obtained from all seven samples (see Tab. N7.1) our experimental data is best fitted with a value of $p_{70} = 0.181$ (see Fig. N7.4). Note that the presented calculation is considering

the highest possible impact from p_{reas} which assumes that from the beginning of the excess measurements all labelled 50S subunits are present to be re-associated into functional 70S ribosomes. It is more probable that this fraction is small in the beginning and will increase over time. This would practically lead to a smaller impact of p_{reas} and finally also to larger p_{70S} values. Although the obtained f_{70S} values exhibit a certain variation within replicates (i.e. measurements with the same excess of unlabeled subunits, but with independent samples, see Fig. N7.4) our major conclusion is clearly verified by the measured data. This conclusion states that we definitely observe a moderate fraction of ribosomes which in protein synthesis makes use of the 70S initiation route. For a further cross-check of our hypothesis we shifted the Cy5 label to the 30S subunit during the translation experiments. The data obtained from both types of samples, i.e. with 30S and with 50S subunit labeling, support our general findings (see Fig. N7.3a,b). Furthermore, we observe overall smaller coincidence fraction for the reference samples in the GFP (i.e. blue) channel as compared, for example, to the dsDNA samples, see Fig. 2b. In the case of an almost complete ribosome subunit labeling with Cy5, the corresponding values would be ~0.9. Therefore, we conclude that the efficiency of subunit labeling (50S and/or 30S) varies from sample to sample and on average gives for samples analyzed here a value of only ~0.6 Cy5 labels per subunit, which is lower compared to what we obtained from absorption measurements (see Methods). However, our major findings are always based on the comparison between the reference sample and the corresponding "excess"- samples (both prepared from the same stock sample) and possible artifacts caused by a lower subunit label ratio are inherently cancelled out.

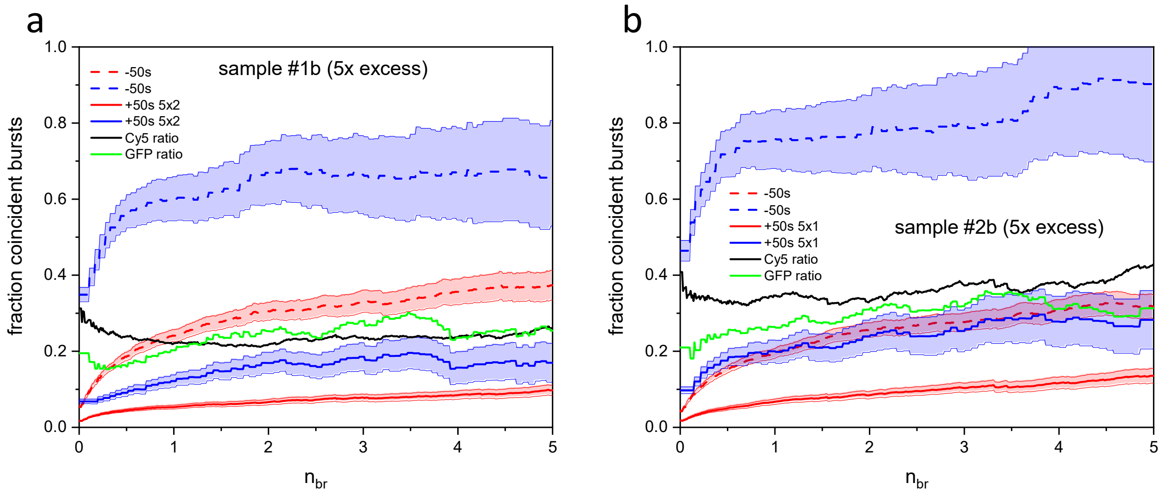

Supplementary Figure N7.1: Coincidence analysis of references samples and 5 fold excess of unlabeled 50S samples. The shaded area around the experimental lines represent the statistical error.

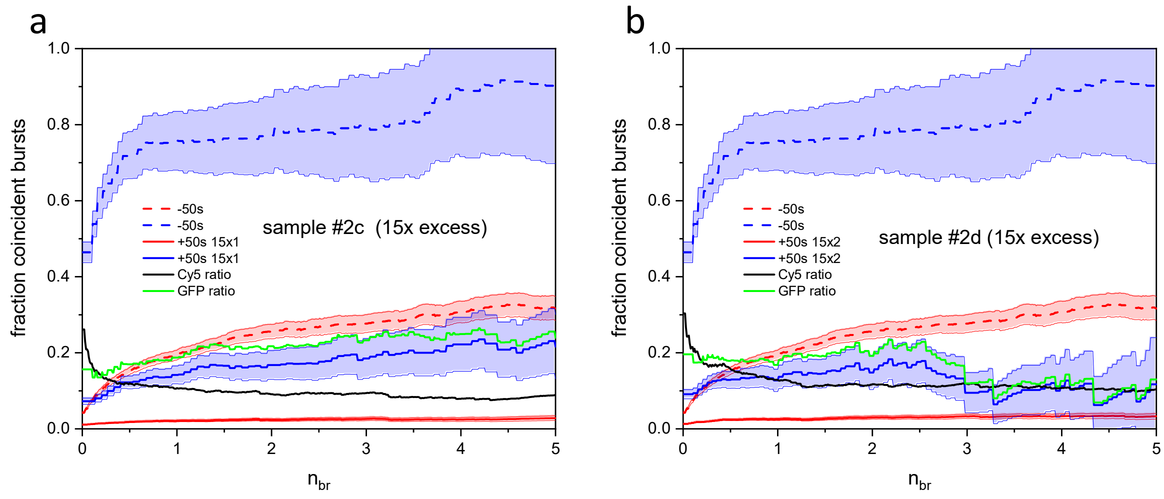

Supplementary Figure N7.2: Coincidence analysis of references samples and 15 fold excess of unlabeled 50S samples. The shaded area around the experimental lines represent the statistical error.

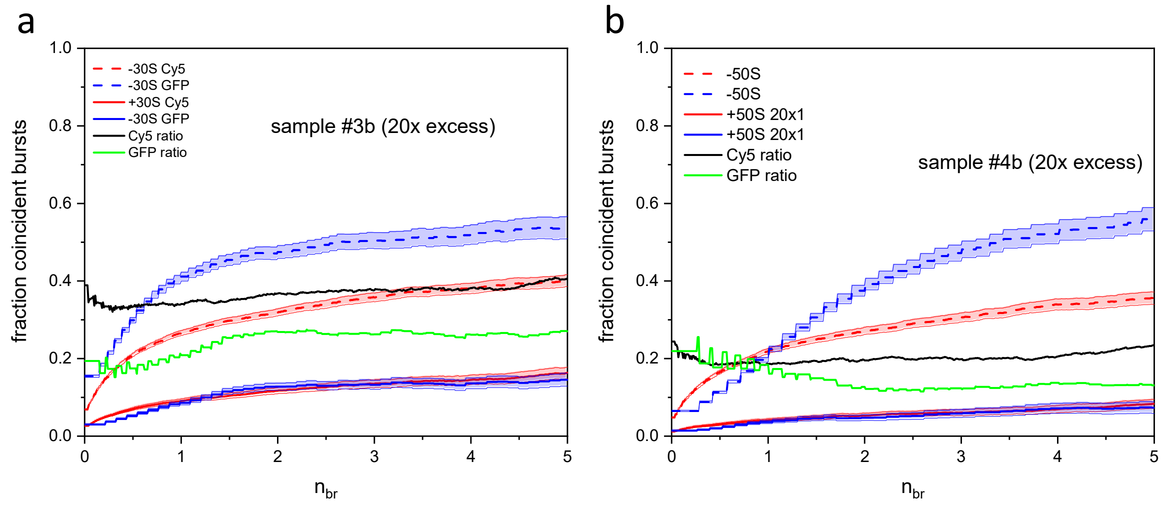

Supplementary Figure N7.3: Coincidence analysis of references samples and 20 fold excess of unlabeled 30S (a) and 50S (b) samples. The shaded area around the experimental lines represent the statistical error.

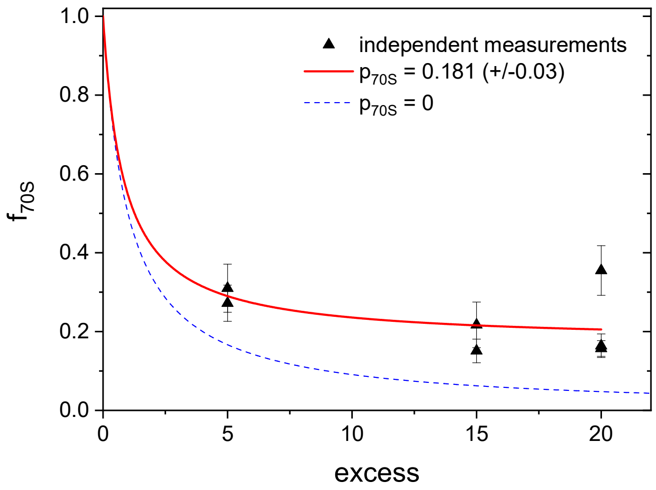

Supplementary Figure N7.4: The red line shows a fit of experimentally determined mean f_{70S} values (black triangles) as obtained at different excess of unlabeled subunits (see Table N7.1) according to eq. 7.4. For comparison a corresponding line (blue dashed) for $p_{70S} = 0$ is shown.

Sample	Excess	GFP channel			Cy5 channel			mean
		Coincidence fraction	Optimal threshold (n_{br})	f_{70s}	Coincidence fraction	Optimal threshold (n_{br})	f_{70s}	f_{70s}
# 1a (ref)	0	0.63 ± 0.06	1.25	-	0.35 ±0 .03	3.62	-	-
# 2a (ref)	0	0.77 ± 0.12	2.70	-	0.25 ± 0.03	3.49	-	-
# 3a (ref)	0	0.51 ± 0.03	3.92	-	0.33 ± 0.02	4.20	-	-
# 4a (ref)	0	0.53 ± 0.03	4.29	-	0.34 ± 0.02	4.33	-	-
#5a (ref)	0	0.56 ± 0.03	3.92	-	0.42 ± 0.02	4.01	-	-
# 1b	5	0.18 ± 0.03	2.23	0.286 ± 0.07	0.09 ± 0.01	4.61	0.257 ± 0.05	0.272 ± 0.046
# 2b	5	0.23 ± 0.05	1.45	0.300 ± 0.11	0.08 ± 0.01	4.20	0.320 ± 0.08	0.310 ± 0.061
# 2c	15	0.14 ± 0.04	1.23	0.181 ± 0.08	0.03 ± 0.01	3.40	0.120 ± 0.05	0.151 ± 0.030
# 2d	15	0.15 ± 0.03	0.78	0.195 ± 0.07	0.06 ± 0.02	3.53	0.240 ± 0.11	0.217 ± 0.058
# 3b	20	0.13 ± 0.02	3.06	0.254 ± 0.05	0.15 ± 0.02	4.49	0.454 ± 0.09	0.355 ± 0.063
# 4b	20	0.08 ± 0.01	4.51	0.151 ± 0.03	0.06 ± 0.01	3.53	0.176 ± 0.04	0.164 ± 0.030
#5b	20	0.10 ± 0.01	2.55	0.179 ± 0.03	0.06 ± 0.01	3.91	0.136 ± 0.04	0.157 ± 0.020

Supplementary Table N7.1: According to the coincidence graphs shown in Figs. N7.1-4 the relevant parameters are given in this table. The errors of the coincidence fractions are determined by the burst statistics and for the f_{70S}-values error propagation was applied. Each sample measurement (with excess x > 0 with) is related to its corresponding reference measurement (with x = 0, given by 1a-5a). The f_{70S} values were calculated according to eq. 7.2. Mean f_{70S} values and the corresponding error of the mean values were determined from f_{70S} values from both channels. Sample #3 was measured with labeled 30S subunits without (#3a) and with 20 fold (#3b) excess of unlabeled 30S. All other samples were measured with labeled 50S subunits with and without excess of unlabeled 50S subunits.

Supplementary Note 8: Comparison of BTCCD to conventional TCCD

We compared the coincidence results of both references (multiple and single labeled) obtained with our BTCCD with that obtained with conventional TCCD. In conventional TCCD the optimal burst threshold for photons in both channels is selected by plotting the association quotient Q as a function of both thresholds and identifying the maximum of Q[6]. The corresponding plots of the DNA origami and two dsDNA reference samples are shown in Supplementary Figures N8a and N8b,c, respectively. Briefly, we used the same data as for BTCCD and binned it into 1ms intervals. We performed the analysis as described in reference[6] and obtained the results given in Supplementary Table N8. We clearly observe underestimated coincidence fractions in comparison to the BTCCD results in the main text.

Sample	C [Hz]	E [Hz]	A [Hz]	B [Hz]	Q	f_{RB}	f_{BR}
DNA nano-beads, origami	2.16	0.01	2.78	2.63	0.66	0.77	0.81
dsDNA(Al488/Al647)	0.92	0.009	1.46	1.57	0.43	0.62	0.58
dsDNA(Al488/Atto647N)	7.28	0.12	11.1	11.0	0.48	0.66	0.66

Supplementary Table N8: Results of conventional TCCD analysis. C is the rate of coincidence events, E is the rate of chance coincidence events, A and B are the rates of events in the red and blue channel, respectively. Q is the association quotient and f_{RB} and f_{BR} are the fractions of coincident red and blue events, respectively.

The extent of underestimation ranges from 0.23 for the DNA nano-beads to 0.34 for the dsDNA (Alexa 488/Atto 647N) reference sample.

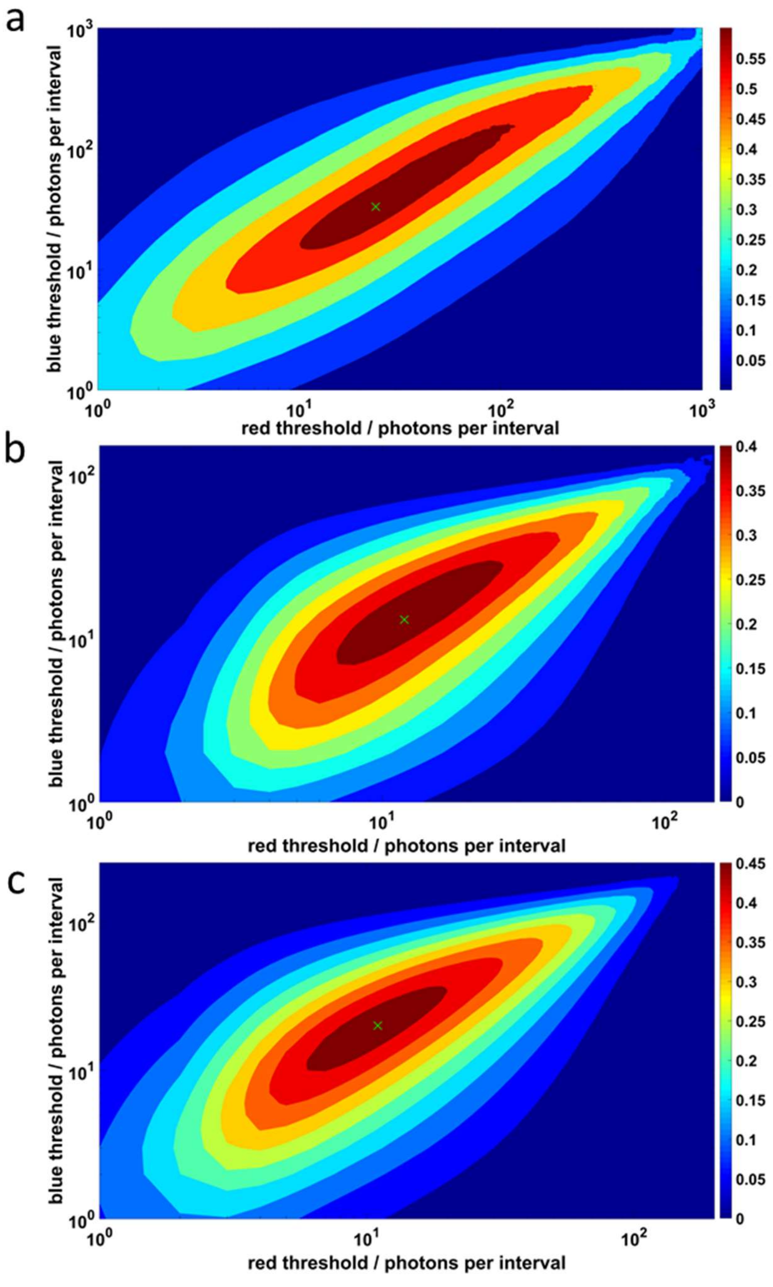

Supplementary Figure N8: Association quotient Q (color code) as a function of conventional TCCD thresholds for red and blue channel shown for (a) multiple labeled DNA-origami nano-bead structure, (b) single-labeled dsDNA (Alexa 488/Alexa 647) and (c) single-labeled dsDNA (Alexa 488/Atto 647N). The position of maximal Q is indicated by a green cross.

Supplementary Note 9: Minimal measurement time of BTCCD experiment

In order to investigate how long a BTCCD measurement needs to be for a reliable quantification of the coincidence fractions, the data obtained for the dsDNA (Alexa 488/Atto 647N) was chopped into subsets that correspond to measuring times of 30 sec., 1 min., 2 min., 5 min. 20min., 2 hrs., 4hrs., and 8 hrs. The rate of detected bursts was 13.5 Hz. Each of these data sets was then individually analyzed with BTCCD.

Supplementary Figure N9.1 shows the coincidence fractions as a function of brightness threshold for the red (a) and blue channel (b). While the red coincidence fractions for 30 sec. and 1 min. are slightly lower than that of longer measuring times, all blue coincidence fractions are similar. For longer measuring times, the noise of the coincidence curves is obviously reduced and for measuring times exceeding 20 minutes. the curves are almost identical. Applying the procedure to identify the optimal brightness threshold (see Suppl. Note 5), one obtains the saturating coincidence fractions given in Supplementary Figure N9.2a. Two features can be observed: (i) the errors for short measuring times are rather large due to the low number of bursts and (ii) the coincidence fractions are underestimated for short measuring times and increase for increasing measuring times until they reach a constant value for measuring times of 20 minutes and longer. The underestimation of the coincidence fractions for short measuring times results from the algorithm to find the optimal brightness threshold (see Suppl. Note 5). If only a low number of bursts is available to calculate the coincidence fraction, the statistical error is high and the determined value of the optimal brightness threshold is at a position where the coincidence fraction did not yet saturate. This can also be seen by comparing the upper ends of the error bars for the low measuring times. They all agree with the saturating coincidence fractions for measuring times larger than 20 minutes. Based on that observation, we performed an empirical fitting of the coincidence fractions as a function of brightness threshold using

$$f_{coinc} = f_{sat} - \Delta f * e^{-n_{br}/n_{1/2}}$$

where f_{sat} is the saturating coincidence fraction, Δf is the increase of the coincidence fraction and $n_{1/2}$ is the half-value brightness threshold.

The obtained f_{sat} values for the different measuring times are given in Supplementary Figure N9.2b. Even for the shortest measuring times, the coincidence fractions are well determined, however, the errors are underestimated. For measuring times of 20 minutes and larger, the fitted coincidence

fractions agree well with that using the optimal brightness threshold (cf Suppl. Fig. N9.2a and N9.2b).

In summary, for short measuring times (<20 minutes) the coincidence fractions using the optimal brightness threshold are underestimated and the saturating coincidence fractions should be determined by eye or by fitting. In that case even a 1 minute BTCCD measurement can provide an accurate value of the coincidences, however, the error due to the low number of analyzed bursts is considerably high (low precision). How long a BTCCD measurement should take depends on the precision that one wants to achieve. A twenty minutes measurement can already provide a reasonable precision.

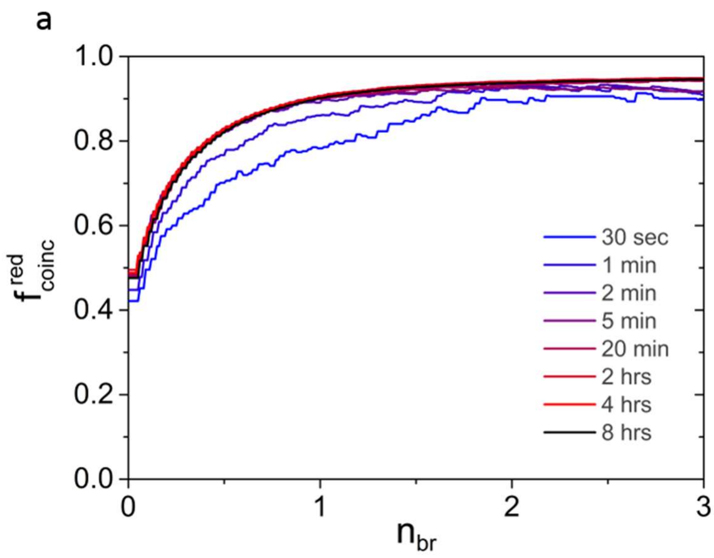

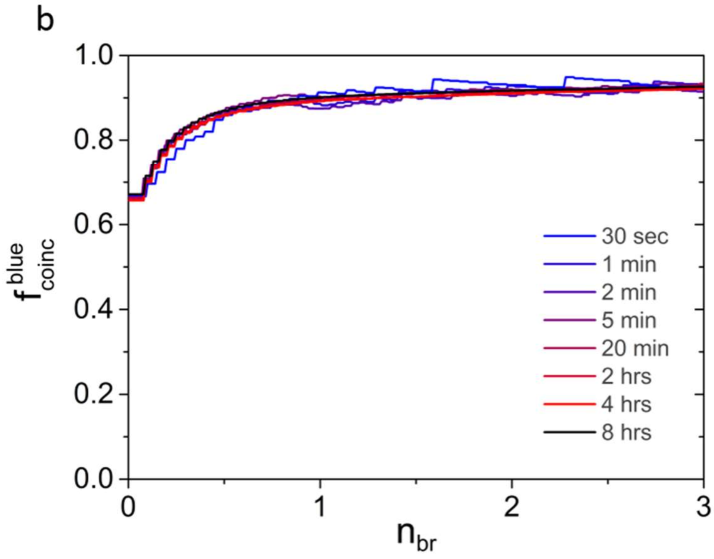

Supplementary Figure N9.1: Fraction of coincident red (a) and blue (b) bursts as a function of brightness threshold for single-labeled dsDNA (Alexa 488/Atto 647N). The measuring time is increased from 30 seconds (blue line) to 4 hours (red line). The full data set was measured for 8 hours (black line). No remarkable differences of the curves can be observed for measuring times of 20 minutes and longer.

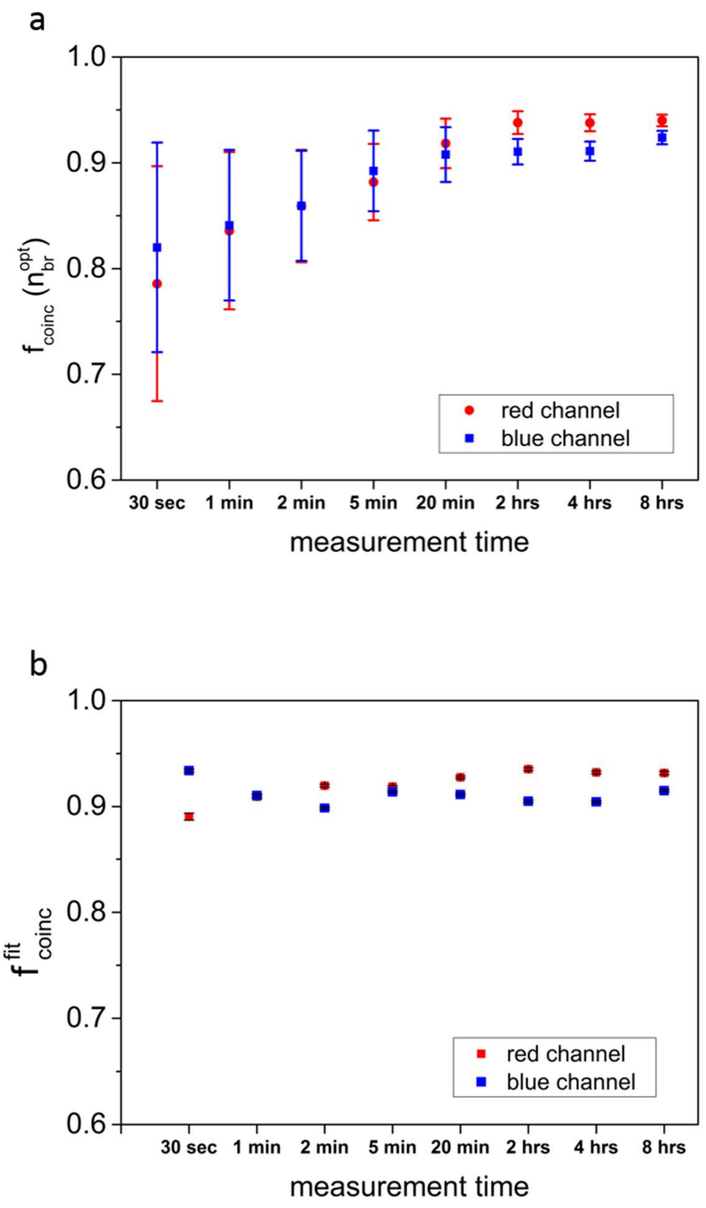

Supplementary Figure N9.2: Saturating coincidence fractions obtained by (a) determination of the optimal brightness threshold (see Suppl. Note 5) or by (b) empirical fitting of coincidence fraction.

References

1. Hoffmann A*, et al.* Quantifying heterogeneity and conformational dynamics from single molecule FRET of diffusing molecules: recurrence analysis of single particles (RASP). *Phys Chem Chem Phys* **13**, 1857-1871 (2011).

2. Hofig H, Otten J, Steffen V, Pohl M, Boersma AJ, Fitter J. Genetically Encoded Forster Resonance Energy Transfer-Based Biosensors Studied on the Single-Molecule Level. *ACS Sens* **3**, 1462-1470 (2018).

3. Liu BQ, Aberg C, van Eerden FJ, Marrink SJ, Poolman B, Boersma AJ. Design and Properties of Genetically Encoded Probes for Sensing Macromolecular Crowding. *Biophys J* **112**, 1929-1939 (2017).

4. Orte A, Clarke RW, Kienerman D. Fluorescence Coincidence Spectroscopy for Single-Molecule Fluorescence Resonance Energy-Transfer Measurements. *Anal Chem* **80**, 8389-8397 (2008).

5. Deuschle K, Okumoto S, Fehr M, Looger LL, Kozhukh L, Frommer WB. Construction and optimization of a family of genetically encoded metabolite sensors by semirational protein engineering. *Protein Science* **14**, 2304-2314 (2005).

6. Clarke RW, Orte A, Klenerman D. Optimized threshold selection for single-molecule two-color fluorescence coincidence spectroscopy. *Anal Chem* **79**, 2771-2777 (2007).
