# Supplementary material for: Brightness-gated two-color coincidence detection unravels two distinct mechanisms in bacterial protein translation initiation
Source: Commun Biol. 2019 Dec 6;2:459. doi: 10.1038/s42003-019-0709-7 (PMC6897966; doi:10.1038/s42003-019-0709-7)
Supplement: Supplementary file 2 — Description of Additional Supplementary Materials [file 42003_2019_709_MOESM2_ESM.docx]

Description of Additional Supplementary Data Files

Supplementary Data 1: Source data underlying plots in Figure 1b.

Supplementary Data 2: Source data underlying plots in Figure 1c.

Supplementary Data 3: Source data underlying plots in Figure 1d.

Supplementary Data 4: Source data underlying plots in Figure 2a.

Supplementary Data 5: Source data underlying plots in Figure 2b.

Supplementary Data 6: Source data underlying plots in Figure 2c.

Supplementary Data 7: Source data underlying plots in Figure 3c.

Supplementary Data 8: Source data underlying plots in Figure 3d.
